# The association between muscle mass and change in physical functioning in older adults: a systematic review and meta-analysis of prospective studies

**DOI:** 10.1007/s41999-025-01230-y

**Published:** 2025-05-23

**Authors:** Marjolein Visser, Katri Sääksjärvi, George L. Burchell, Laura A. Schaap

**Affiliations:** 1 Department of Health Sciences, Faculty of Science, Vrije Universiteit Amsterdam, Van der Boechorststraat 3, 1081 BT Amsterdam, The Netherlands; 2 Amsterdam Public Health Research Institute, Amsterdam, The Netherlands; 3 Department of Public Health, Finnish Institute for Health and Welfare, Helsinki, Finland; 4 Medical Library, Vrije Universiteit Amsterdam, Amsterdam, The Netherlands; 5 Amsterdam Movement Sciences, Vrije Universiteit Amsterdam, Amsterdam, The Netherlands

**Keywords:** Sarcopenia, Functional decline, Aging

## Abstract

**Purpose:**

The aim of this review is to investigate the prospective association between muscle mass and physical functioning in older adults, and to explore sources of variability in this association.

**Methods:**

Three databases were searched for prospective, observational studies investigating any assessment of muscle mass and functional decline in older adults (mean age 65 + years). 72 studies were included. Meta-analyses using results of the most adjusted statistical models were performed using Review Manager version 5.3.5.

**Results:**

Most studies were conducted in the community setting (*n* = 64) and used incident ADL limitations/disability as outcome (*n* = 44). Across studies, 6 body composition methods and 34 different parameters of muscle mass were used, including ratios. Average follow-up time was 46 months. For low versus higher muscle mass (*n* = 25), the overall odds ratio (OR) for functional decline was 1.23 (95% CI 1.10–1.37, *I*^2^ = 70%). Per SD higher muscle mass (*n* = 15), the overall OR was 0.89 (0.83–0.96, 76%). In general, no associations were observed when no adjustment for body size, or for body height only, was made. Associations were generally stronger when muscle mass was expressed as ratio of body weight, BMI or fat mass, or when more accurate body composition methods or objective performance tests were used.

**Conclusion:**

In contrast to earlier reviews, this updated review shows that lower muscle mass is associated with higher risk of functional decline in older adults. Earlier observed variability in this association is explained by body composition method, body size adjustment, and type of physical functioning outcome.

**Supplementary Information:**

The online version contains supplementary material available at 10.1007/s41999-025-01230-y.

## Key summary points

**Aim:**

To investigate the prospective association between lower muscle mass and physical functioning in older adults.

**Findings:**

Overall, lower muscle mass increases the risk of functional decline by about 25%. Earlier variability observed in this association can be explained by body composition method, body size adjustment, and type of physical functioning outcome.

**Message:**

In contrast to earlier reviews, this updated review clearly shows that lower muscle mass is associated with higher risk of functional decline in older adults.

**Supplementary Information:**

The online version contains supplementary material available at 10.1007/s41999-025-01230-y.

## Introduction

Low muscle mass in older adults is generally regarded as an important risk factor for negative clinical outcomes [1–3]. The role of low muscle mass in accelerating functional decline remains, however, controversial. Our systematic review and meta-analysis published 12 years ago concluded on the basis of six prospective studies that muscle mass was not associated with functional decline [4]. Prospective studies have also shown that low muscle strength (i.e., handgrip strength) is more strongly associated with negative health outcomes as compared to low muscle mass [5–7]. Because of these findings and the challenges of measuring muscle mass, especially in the community setting, some recent sarcopenia consensus groups have placed a greater emphasis on low muscle strength instead of low muscle mass [8–10].

In recent years, the number of published studies on the association between muscle mass and subsequent change in physical functioning has drastically increased, warranting an update of our review. This larger number now also allows the investigation of factors potentially influencing the association, in order to explore reasons for the high heterogeneity previously observed [4].

A first factor that may influence the association between muscle mass and change in physical function is the body composition method used to assess muscle mass. Simple anthropometric assessments such as calf circumference and estimates of muscle mass from prediction equations may not accurately reflect whole-body skeletal muscle mass. Discussions regarding more advanced methods to accurately assess whole-body skeletal muscle mass are also ongoing [11–13]. Use of relatively inaccurate muscle mass assessment methods may underestimate the true association between muscle mass and change in physical functioning.

A second factor is how body size is taken into account in the studies investigating the association between muscle mass and change in physical functioning. This includes body height (taller people generally have greater muscle mass [14]) and body weight (people with a higher body weight, BMI, or fat mass generally have greater muscle mass [15]). As both body height and body weight are also associated with physical functioning [16, 17], they should be accounted for when examining the association between muscle mass and change in physical functioning. Previous studies used different approaches, such as no adjustment for body size, using muscle mass ratios (e.g., muscle mass divided by height squared, or muscle mass divided by body weight), including body size (e.g., height and/or body weight) as confounder in the statistical models, or using muscle mass residuals derived from the regression on body height and fat mass. It remains unclear what the impact of these different approaches is on the association between muscle mass and change in physical functioning.

A final factor that may influence the results is the method used to assess change in physical functioning. This can include an objective measurement of physical functioning using a single performance test or a battery of tests, as well as measures of self-reported physical functioning, such as self-reported mobility, activity of daily living (ADL) or instrumental ADL (IADL) limitations or disability.

The primary aim of this systematic review was to provide an up-to-date overview of prospective studies investigating the association between muscle mass and functional decline in older adults. The secondary aim was to explore the potential role of body composition methodology, body size adjustment, and type of physical functioning outcome in this association.

## Methods

This review is registered in PROSPERO (identifier CRD42023450696).

### Search strategy

A systematic search was performed in the databases: PubMed, Embase.com and Clarivate Analytics/Web of Science Core Collection. The timeframe within the databases was from inception to 25 th October 2024 and conducted by GLB. The search included keywords and free text terms for (synonyms of) ‘muscle mass’ combined with (synonyms of) ‘functional decline’ combined with (synonyms of) ‘older population’. A full overview of the search terms per database can be found in the supplementary information (see Annex 1). No limitations on date or language were applied in the search.

### Screening

The screening of titles and abstract for eligibility was conducted by one reviewer (LS or KS) and incorporated machine learning [18]. The prospective papers included in our previous review reporting on the association between muscle mass and change in physical functioning [4] and several irrelevant papers (*n* = 5) were added to ASReview as the basis for machine learning (‘prior knowledge’). A data-driven approach was used in which the screening process was stopped after screening 20% of the total number of papers, combined with subsequent 5% classification of irrelevant papers [19]. Once titles and abstracts were screened, full texts of the included references were uploaded into the Covidence systematic review software system (Covidence, Melbourne, Australia) and screened by two reviewers independently for inclusion (MV and KS). Reviewers were blinded for each other’s decisions while screening. Disagreements were first discussed by the two reviewers, and if no consensus could be derived, a third reviewer (LS) made the decision. In a final step, backward snowballing was used. Reference lists of 10 randomly selected included studies were checked for additional studies (KS). The combination of machine learning and backward snowballing has been shown to achieve better results than traditional screening [20].

Inclusion criteria were:

1. Language: only publications written in English were considered.
2. Participants: older adults (mean age at 65 years or more) in any setting (community, hospital, institution). These studies may include older adults with diseases or conditions.
3. Types of studies: prospective, longitudinal observational studies (cohort and case–control studies).
4. Exposure: at least one baseline assessment of ‘muscle mass’, which includes but is not limited to muscle mass, lean mass, fat-free mass, muscle cross-sectional area, muscle density, muscle circumference, muscle thickness, and muscle volume.
5. Outcome: change in physical functioning, either objectively assessed (e.g., gait speed or short physical performance battery) or self-reported (e.g., mobility limitations and Barthel Index).
6. Publication type: result paper.

Exclusion criteria were:

1. Studies that focus on older adults from one specific patient group only (e.g., older adults with hip fracture, diabetes, or cancer).
2. Studies that used muscle mass only in combination with other parameters such as grip strength or physical functioning in order to determine sarcopenia (for example by applying the EWGSOP algorithm).
3. Conference abstracts, conference proceedings.
4. Not peer-reviewed publications (such as editorials).

When a reference was excluded, the reason for exclusion was indicated using the following fixed hierarchy to minimize potential conflicts: 1) full text not available, 2) wrong type of publication (e.g., conference abstract or conference proceeding), 3) wrong study type (e.g., cross-sectional study or intervention study), 4) wrong population (e.g., not meeting age criterion or specific patient group only), 5) wrong exposure (e.g., fat mass or total body water, or sarcopenia using an algorithm), and 6) wrong outcome (e.g., incident frailty or change in quality of life).

### Data extraction

The following data from the included studies were extracted by one author (MV) and recorded in a standardized data extraction Excel spreadsheet: bibliographic information (first author and year of publication), study design, cohort name, country of study participants, sample size, sex, used body composition methodology to estimate muscle mass, the specific parameter(s) of muscle mass (change) including all ratios or ways to express muscle mass, physical functioning outcome(s), follow-up time(s), effect size(s) of the association(s) with their measure of variation and confounders. The specific parameter of muscle mass was extracted after carefully reading the methods section of a study (including provided references related to validation studies or prediction equations) and applying the Global Leadership Initiative on Sarcopenia glossary [21], and not the original authors’ terminology. The muscle mass parameter abbreviations used in this paper are shown in Table 2 of “Results”. Effect size of the most adjusted model (regression coefficient, odds ratio, risk ratio, or hazard ratio) was extracted for all muscle mass parameters and all physical functioning outcomes from a single study. When models with and without a measure of body size as confounder were reported, both effect sizes were extracted. The data extraction was checked by a second author (LS) based on a 10% random sample, and deviations were discussed and solved.

### Risk of bias assessment

The assessment was done at study level using eight criteria of the CLARITY scale for cohort studies [22, 23]. As suggested by Grooten et al. [24], the three authors first discussed how to judge these criteria in advance and performed a pilot test based on ten included studies. The eighth criterion, “Were co-interventions similar between groups?” was considered not applicable and replaced with a new criterion: “Were there any other flaws in this study?” After a final revision (Annex 2), the quality assessment was performed by one reviewer (LS or KS), and a random selection of 20 studies was checked by the other reviewer. Disagreements were reviewed by a third reviewer (MV) who made the final decision. Risk of bias criteria 1–7 were scored as: definitely no = 0, probably no = 1, probably yes = 2, definitely yes = 3. This led to a final score per study ranging from 0 to 21. Studies were scored as high risk of bias when at least two criteria were scored as ‘definitely no’, or one criterion with ‘definitely no’ and two criteria with ‘probably no’, or three criteria with ‘probably no’. Studies having a (major) flaw (criterion 8) were also scored as high risk of bias. Some risk of bias was defined as having one or two criterion scores as ‘probably no’. When all criteria were scored as ‘probably yes’ or ‘definitely yes’, the study was considered as low risk of bias [25].

### Data analysis

Descriptive characteristics of the included studies were analyzed. Meta-analyses were performed using Review Manager 5.3, version 5.3.5 [26] using a random-effects model. Two meta-analyses were carried out for studies that investigated the association between baseline muscle mass and a dichotomous physical function outcome (decline versus no decline). The first meta-analysis included studies that used baseline muscle mass as a dichotomous variable (low versus not low) or as a categorical variable (e.g., tertiles, quartiles or quintiles). For a dichotomous variable, the effect size for the low muscle mass group versus the not-low group was used. In the case of a categorical variable, the effect size of the lowest muscle mass category versus the highest category was used. Effect sizes were reversed when the reference group included those with low muscle mass instead of those with high muscle mass. The second meta-analysis included studies that used baseline muscle mass as a continuous variable. The effect sizes per standard deviation (SD) higher muscle mass were used. Effect sizes were reversed when reported per unit or per SD lower muscle mass. When the effect size was reported per unit of muscle mass, the reported standard deviation of baseline muscle mass was used to calculate the effect size per SD.

For both meta-analyses, the reported effect size for men and women combined was used. In addition, the analyses were performed for men and women separately. When the effect sizes were reported for men and women separately in a single study, the pooled effect size was calculated using a fixed effect model. Several studies were based on data from the same cohort study, such as the Health, Aging and Body Composition study, the MrOS studies in the United States of America and in Hong Kong, and the Cardiovascular Health Study. Only one study from a cohort was used in a single meta-analysis (depending on the selection criteria for that meta-analysis), in general the most recent study with the longest follow-up.

The effect sizes of the studies were pooled using the random-effects model that resulted in forest plots. Heterogeneity was evaluated by the *I*^2^ statistic, which expresses the percentage of variation attributable to between-study heterogeneity. An *I*^2^ of 50% or higher was interpreted as substantial heterogeneity. All *p* values were 2 tailed (*α* = 0.05). Funnel plots were created to inspect potential publication bias.

In both meta-analyses, potential effect modification was explored using additional meta-analyses stratified by the following subgroups:

- i. Objective functional performance measure; ii. self-reported mobility limitation or disability; iii self-reported I(ADL) limitation or disability as outcomes;
- i. Relatively high accuracy methods (DXA, (pq)CT, D_3_Cr); ii. relatively low accuracy methods (BIA, anthropometry);
- i. No adjustment for body size or using muscle mass/height squared ratio; ii. using ratio of muscle mass/weight, muscle mass/fat mass or muscle mass/BMI.

The associations obtained from studies that included physical function as a continuous outcome only, as well as from studies investigating the association between change in muscle mass versus change in physical functioning over time only, were described in separate tables as their results could not be pooled due to large methodological differences. When studies used other statistical tests (e.g., a Student t-test) or did not provide an effect size (e.g., the authors stated in the text that the association was not statistically significant), the results were presented in a table and described in the text. All studies were considered when formulating conclusions about the overall association between muscle mass and change in physical functioning.

## Results

**Fig. 1:**
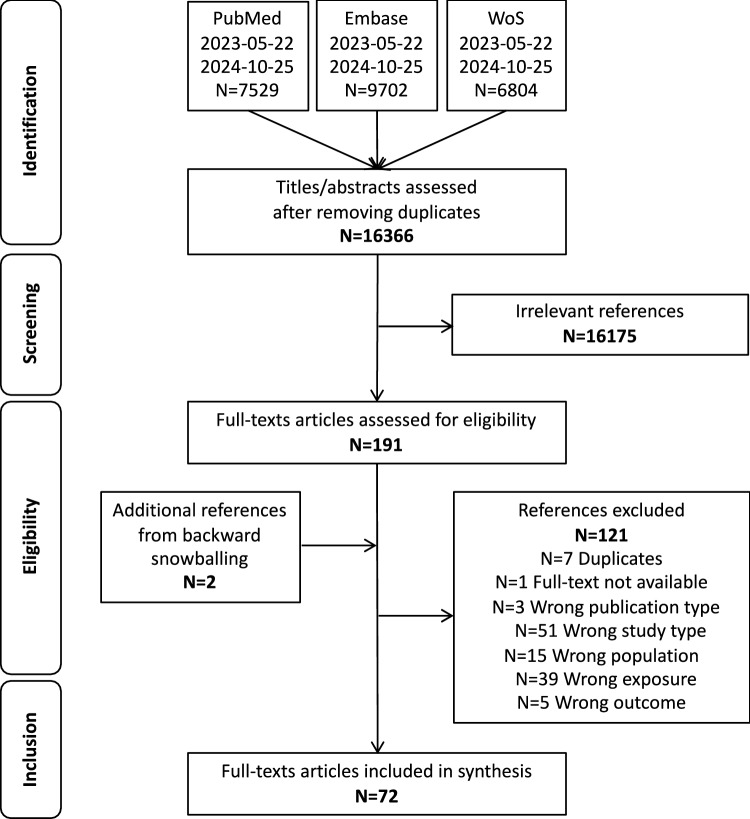
PRISMA flow diagram of study selection

From the 16,366 identified references after excluding duplicates, 191 were included for full article screening (Fig. 1). Of the 191 full articles, and the 2 articles obtained by backward snowballing, 72 articles were included in the review [27–97]. The main characteristics and the risk of bias evaluation of each included study are shown in Annex 3 and Annex 4. Of the 72 included studies, 34 were scored at low risk of bias, 35 at some risk of bias, and 4 at high risk of bias [31, 64, 84, 86].

Some descriptive characteristics of the included studies are shown in Table 1. The majority of studies were conducted in the community setting (64 studies) and mainly included older adults living in the Unites States of America (29 studies), Japan (10 studies) and Italy (7 studies). The mean follow-up time to assess change in physical functioning was 46 months, with a range from 2 to 168 months. Most studies used self-reported measures of functional limitation, such as ADL (*n* = 44), mobility (*n* = 21) and IADL (*n* = 11) limitations or disability. Seventeen studies used one or more performance tests as study outcome. Many studies included more than one physical functioning outcome.

**Table 1:** General characteristics of the *n* = 72 included studies investigating the association between muscle mass and change in physical functioning in older adults

Characteristic	*N* of studies
Setting	
Community	64
Hospital	6
Nursing home	2
Methodology used to assess muscle mass	
Dual-energy X-ray absorptiometry (DXA)	38
Bioelectrical impedance (BIA)^1^	21
(Peripheral quantitative) computer tomography ((pq)CT)	8
D3-Creatinine dilution (D3 Cr)	7
Anthropometry^2^	7
Ultrasound (US)	2
Outcomes used to assess change in physical functioning	
Activities of daily living limitations or disability (ADL)	44
Mobility limitations or disability	21
Instrumental ADL limitations or disability	11
Performance test(s)	17
Follow-up time for outcome assessment (months)	Mean (min–max)
All studies (*n* = 69)^3^	46 (2–168)
Community (*n* = 62)	50 (6–168)
Hospital (*n* = 5)	5 (2–12)
Nursing home (*n* = 2)	9 (6–12)

Study specific details can be found in Annex 3

^1^Includes two studies using bio-impedance spectroscopy (BIS)

^2^Includes calf circumference, corrected arm muscle area, or skeletal muscle mass predicted from multiple anthropometric measures

^3^For three studies, no follow-up time was reported

The most frequently used body composition methods to assess muscle mass were dual-energy X-ray absorptiometry (38 studies) and bioelectrical impedance (21 studies). Overall, 34 different muscle mass parameters were used across the included studies (Table 2), with 29 studies including more than one parameter. Some of this variation in parameters was due to the body composition method to assess muscle mass, and some was due to how a muscle mass parameter was expressed (e.g., in absolute kg or divided by another body parameter). Most studies included some kind of appendicular lean soft tissue mass parameter (ALM, *n* = 44), followed by some kind of cross-sectional muscle area parameter (CSA, *n* = 9), whole-body skeletal muscle mass (SMM, *n* = 9) or total body lean soft tissue mass parameter (LM, *n* = 8). The most frequently used muscle parameters across all studies were appendicular lean soft tissue mass divided by body height squared (ALM/ht^2^, *n* = 27), ALM divided by BMI (ALM/BMI, *n* = 15) and ALM in kg (*n* = 11).

**Table 2:** Overview of the muscle mass parameters used in the relevant statistical models of the included studies investigating the association between muscle mass and change in physical functioning in older adults

Muscle parameter	Unit	Abbreviation used for this review	Number of studies
Appendicular lean soft tissue mass divided by body height squared	kg/m^2^	ALM/ht^2^	27
Appendicular lean soft tissue mass divided by body mass index	kg/kg/m^2^	ALM/BMI	14
Appendicular lean soft tissue mass	kg	ALM	12
Appendicular lean soft tissue mass divided by body weight	kg/kg	ALM/Wt	7
Skeletal muscle mass divided by body height squared	kg/m^2^	SMM/ht^2^	7
Muscle mass divided by body weight	kg/kg	MM/Wt	6
Muscle cross-sectional area at the mid-thigh	cm^2^	CSA thigh	5
Skeletal muscle mass	kg	SMM	4
Fat-free mass	kg	FFM	4
Lean soft tissue mass divided by body fat mass	kg/kg	LM/FM	3
Lean soft tissue mass	kg	LM	3
Muscle mass	kg	MM	3
Muscle cross-sectional area at 66% of tibia length	cm^2^	CSA tibia	3
Calf circumference	cm	CC	2
Leg lean soft tissue mass	kg	Leg LM	2
Residual of appendicular lean soft tissue mass regressed on body height and body fat mass	kg	ALM residual	2
Bilateral anterior thigh thickness	cm	BATT	2
Appendicular lean soft tissue mass divided by body height squared, divided by body fat mass divided by body height squared	kg/m^2^/kg/m^2^	ALM/ht^2^/FM/ht^2^	2
Phase angle	degrees	PhA	2
Corrected arm muscle area	cm^2^	CAMA	1
Skeletal muscle mass divided by body weight	kg/m^2^	SMM/Wt	1
Skeletal muscle mass as a percentage of body weight	%	SMM%	1
Body fat mass divided by leg lean soft tissue mass	kg/kg	FM/Leg LM	1
Body fat mass divided by fat-free mass	kg/kg	FM/FFM	1
Body weight divided by fat-free mass	kg/kg	Wt/FFM	1
Fat-free mass as a percentage of body weight	%	FFM%	1
Appendicular fat-free mass	kg	AFFM	1
Leg fat-free mass	kg	Leg FFM	1
Muscle cross-sectional area at L4-L5 level	cm^2^	CSA trunk	1
Leg lean soft tissue mass divided by leg length squared	kg/m^2^	Leg LM/Leght^2^	1
Appendicular lean soft tissue mass divided by body fat mass	kg/kg	ALM/FM	1
Body cell mass	kg	BCM	1
Calf segmental intracellular resistance skeletal muscle index	cm^2^/Ohm	Cri-SMI	1
Muscle mass divided by body height squared	kg/m^2^	MM/ht^2^	1

### Meta-analysis 1—low baseline muscle mass and decline in physical function

Of the majority of studies that examined the association between baseline muscle mass and subsequent change in physical functioning as a dichotomous variable (decline versus no decline), *n* = 36 contrasted low muscle mass with non-low muscle mass at baseline. These studies were the basis for the first meta-analysis. Data from six studies were not used due to cohort overlap [5, 27, 30, 42, 43, 72], and data from three studies [40, 60, 90] were only used in a stratified meta-analysis due to cohort overlap. Of the studies that were (partially) included, most studies (*n* = 18) compared those with low muscle mass with those having a non-low muscle mass, and none had a high risk of bias.

A large variety in the applied cutoff point to determine low muscle mass was observed. These included cutoff points suggested by consensus groups (such as EWGSOP2 [98], AWGS [99] and the revised AWGS [9]), analytically derived cutoff points based on the association with a clinical outcome (such as FNIH [100] or Janssen et al. [101]), a percentile cutoff of the sample itself (such as lowest 20% or 50%), or a cutoff based on the Z score of a reference sample [102–104]. For the other studies, the lowest sample-based tertile (*n* = 1), quartile (*n* = 4) or quintile (*n* = 4) was used to define low muscle mass for this meta-analysis. For one study, the 10 th percentile was used which was compared with a sex-specific reference value [80].

**Fig. 2:**
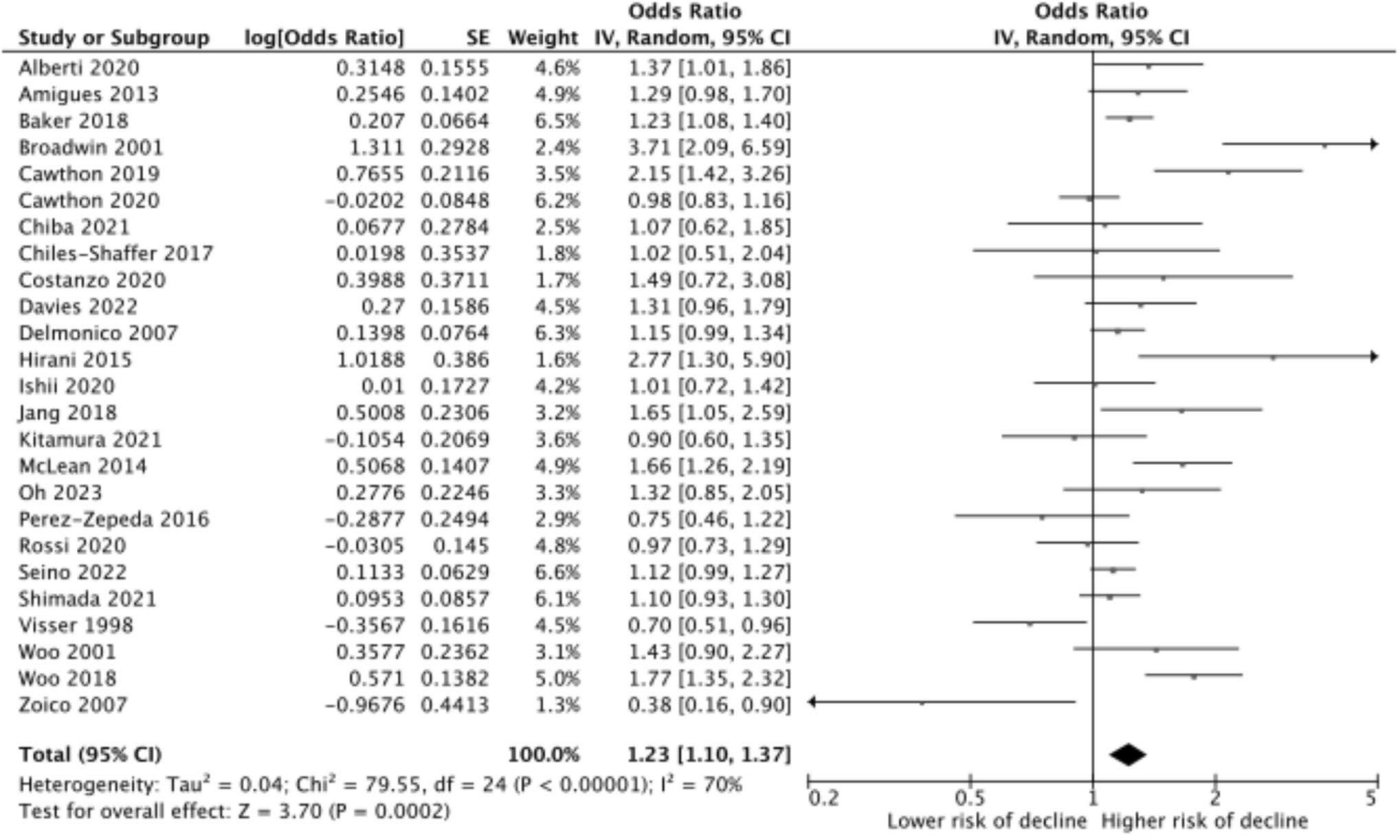
Random-effect meta-analysis of the risk of developing functional decline in older adults with low baseline muscle mass versus those with not-low muscle mass (reference group)

Figure 2 shows the forest plot of the overall effect size based on 25 included studies. As many studies used multiple muscle mass parameters and physical functioning outcomes, the effect size of the most optimal model was selected using the following hierarchy: most accurate body composition method, performance tests over mobility limitation over ADL disability over IADL disability, best adjustment for body size (i.e., included as confounders in regression models) over using muscle mass ratios with weight, BMI or fat mass, over adjustment for height only, over no body size adjustment). None of the included studies had a high risk of bias. Overall, low baseline muscle mass was associated with a 23% higher risk of developing functional decline over time (OR 1.23 (95% CI 1.10–1.37)), with a high level of heterogeneity (*I*^2^ = 70%). Results were OR 1.22 (95% CI 1.02–1.45, *I*^2^ = 62%) for men (based on 13 studies) and OR 1.27 (95% CI 1.04–1.54), *I*^2^ = 76%) for women (based on 12 studies). The funnel plot did not indicate publication bias (Annex 5).

To explore the role of a) the method used to assess the study outcome, b) the accuracy of the body composition method used to assess baseline muscle mass, and c) the method used to adjust for body size, studies were stratified according to these three factors. None of the included studies had a high risk of bias. The forest plots for each of the strata can be found in Annex 6a-6c. The overall effect sizes, with measure of heterogeneity, for all strata are shown in Table 3. With regard to the risk of functional decline in those with low versus not-low muscle mass, the largest effect size (OR 1.61 ((%% CI 1.36–1.91)) was observed for studies using an objective performance test as study outcome, with no heterogeneity (*I*^2^ = 0%). The overall effect size for (I)ADL also was statistically significant with low heterogeneity. The overall effect size for self-reported mobility limitation did not reach statistical significance and heterogeneity remained high. Stratification into two groups according to accuracy of the body composition method used to assess muscle mass did not reduce heterogeneity; however, the overall effect size based on 14 studies using less accurate methods such as BIA and anthropometry was relatively smaller compared to that of the 14 studies using higher accuracy methods and was no longer statistically significant. When studies were meta-analyzed that reported an effect size not adjusted for body size or adjusted for height only, no statistically significant overall association between low muscle mass and decline in physical function was observed. In contrast, when the overall effect sizes based on the ratio of muscle mass/weight, muscle mass/fat mass or muscle mass/BMI were meta-analyzed, the association was strongest (OR 1.49 (95% CI 1.22–1.81)), without reducing heterogeneity. Heterogeneity was lowest (32%) when effect sizes were used from studies that included weight, fat mass, or BMI as a confounder in the regression models. The overall effect size remained statistically significant (OR 1.17 (95% CI 1.07–1.27)).

**Table 3:** Random-effect meta-analyses of the risk of developing functional decline in older adults with low baseline muscle mass versus those with not-low muscle mass, as well as per standard deviation higher baseline muscle mass, categorized by a) type of outcome measure, b) accuracy of the body composition method to assess muscle mass, and **c**) adjustment for body size

Stratification factor	Strata	N of studies	Overall effect size for low muscle mass versus not-low (OR (95% CI))	Heterogeneity (*I*^2^)	N of studies	Overall effect size per SD higher muscle mass (OR (95% CI))	Heterogeneity (*I*^2^)
Physical functioning outcome measure	Objective functional performance measure	5	**1.61 (1.36–1.91)**	0%	1	NA	NA
	Self-reported mobility limitation or disability	7	1.12 (0.81–1.55)	87%	7	**0.84 (0.72–0.97)**	75%
	Self-reported I(ADL) limitation or disability	19	**1.21 (1.12–1.31)**	29%	12	0.92 (0.84–1.01)	79%
Accuracy of body composition method	Relatively high (DXA, (pq)CT, D_3_Cr)	14	**1.26 (1.10–1.45)**	69%	9	**0.86 (0.79–0.93)**	78%
	Relatively low (BIA, Anthropometry)	14	1.18 (0.98–1.43)	72%	6	0.98 (0.81–1.17)	70%
Adjustment for body size	None or using muscle mass/height squared ratio	18	1.07 (0.92–1.24)	75%	8	1.01 (0.92–1.09)	52%
	Using ratio of muscle mass/weight, muscle mass/FM or muscle mass/BMI	10	**1.49 (1.22–1.81)**	76%	5	**0.77 (0.68–0.87)**	76%
	Using height, weight, fat mass, or BMI as confounder in statistical model	11	**1.17 (1.07–1.27)**	32%	8	0.90 (0.81–1.02)	77%

Effect sizes in bold indicate a statistically significant association

ADL activities of daily living, IADL instrumental ADL, DXA dual-energy X-ray absorptiometry, (pq)CT (peripheral quantitative) computer tomography, D3 Cr D3-Creatinine dilution, OR odds ratio, CI confidence interval, *I*^2^ heterogeneity

### Meta-analysis 2—baseline muscle mass (per SD higher) and decline in physical function

**Fig. 3:**
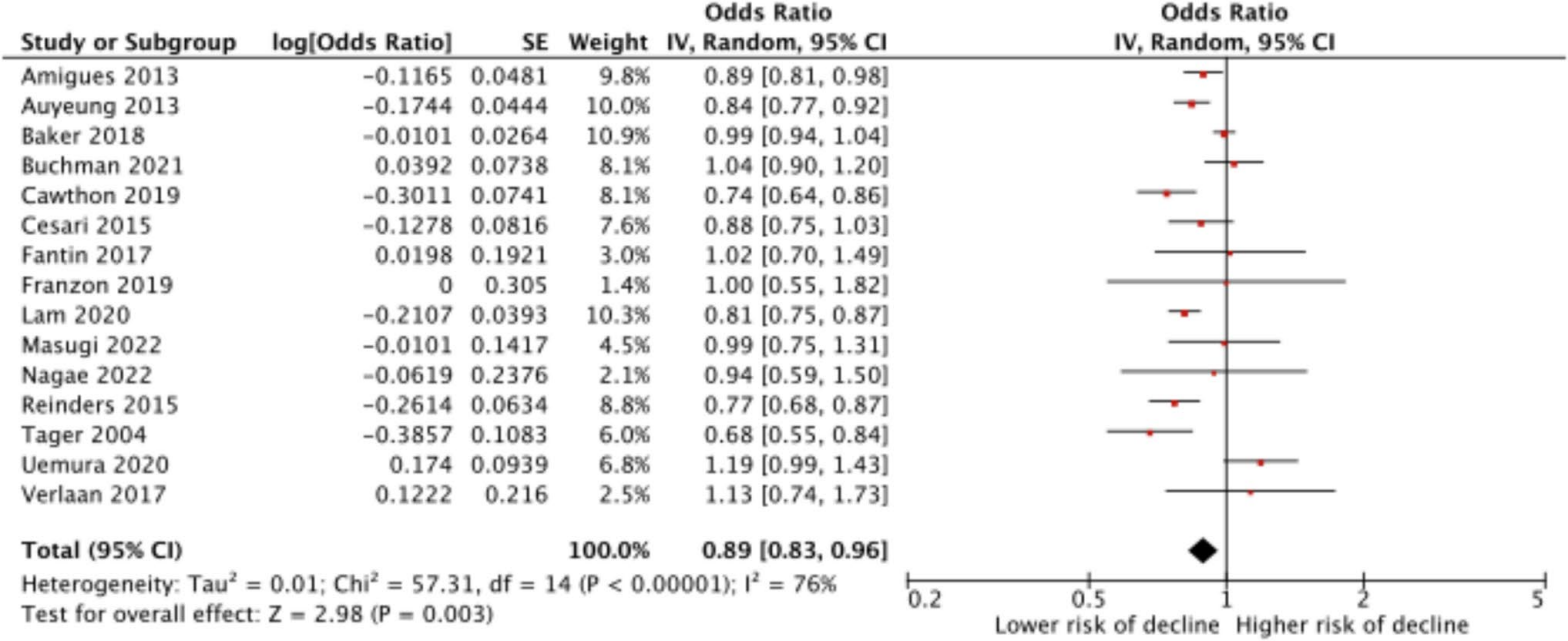
Random-effect meta-analysis of the risk of developing functional decline in older adults per standard deviation higher baseline muscle mass

Nineteen studies used baseline muscle mass as a continuous variable when investigating the association with subsequent change in physical functioning (decline versus no decline). Data from three studies were not used due to cohort overlap [27, 30, 73], and data from one study [42] were only used in a stratified meta-analysis due to cohort overlap. Overall, per standard deviation higher baseline muscle mass, the risk of developing functional decline over time was 11% lower (OR 0.89 (95% CI 0.83–0.96)) with a high heterogeneity (*I*^2^ = 76%) (Fig. 3). Results were OR 0.87 (95% CI 0.83–0.92, *I*^2^ = 82%) for men (based on 9 studies) and OR 0.89 (95% CI 0.82–0.97), *I*^2^ = 72%) for women (based on 8 studies). Two of the included studies had a high risk of bias [31, 86]. Excluding these two studies from the meta-analysis gave similar results (OR 0.89 (95% CI 0.82–0.97), *I*^2^ = 78%). The funnel plot did not indicate publication bias (Annex 5).

The forest plots for all stratified meta-analyses can be found in Annex 7a-7c. As only one study [76] used decline in performance test score (gait speed), no meta-analysis was possible for that study outcome. The overall risk per SD higher muscle mass was lowest and statistically significant when mobility disability was used as the outcome (Table 3). No statistically significant effect size was observed for studies using (I)ADL disability as the outcome. Heterogeneity remained high in both strata. Similar to the stratified results for low versus not-low muscle mass, the overall effect size was stronger and statistically significant only when higher accuracy body composition methods were used to assess muscle mass instead of lower accuracy methods. Associations were strongest when muscle mass ratios with weight, fat mass or BMI as the denominator were used and weakest (and not statistically significant) when muscle mass was used as an absolute measure or divided by height squared without any other body size adjustment.

Six studies investigated the association between baseline muscle mass and change in physical functioning using a continuous outcome measure. Because of their continuous outcome, these studies were not included in the previous meta-analyses, and large methodological differences between studies hampered a separate meta-analysis. Their main results are, therefore, shown in Table 4 (further details of these studies can be found in Annex 3). All six studies showed at least one statistically significant association between baseline muscle mass and change in physical functioning, although within studies differences were observed between men and women [35, 67], muscle parameters [35, 37, 55, 67, 71], and physical functioning outcomes [67].

**Table 4:** Overview of the six studies investigating the association between baseline muscle mass in older adults and change in a continuous measure of physical functioning

First author	Publication year	Body size adjustment	Most adjusted effect size	Statistically significant association (lower baseline muscle mass is associated with greater decline in physical function)
Beavers	2013	None	B (SE, *p*-value) per SD higher baseline muscle mass Males: LM 0.0005 (0.0067, *p* = 0.94), ALM 0.0081 (0.0067, * p* = 0.22); legLM 0.0065 (0.0063, * p* = 0.3); CSA_thigh_ 0.0113 (0.0062, * p* = 0.07) Females: LM −0.0267 (0.0083, p < 0.01); ALM −0.0274 (0.0081, p < 0.01); legLM −0.026 (0.0071, p < 0.01); CSA_thigh_ 0.0044 (0.0062, * p* = 0.47)	Gait speed LM males no, females yes ALM males no, females yes Leg LM males no, females yes CSA_thigh_ males no, females no
Björkman	2019	BMI as confounder	B (95% CI) per SD higher baseline muscle mass SMM/ht^2^: −0.01 (−4.37–4.10, * p* = 0.951) Cri-SMI: 0.15 (1.07–18.63, * p* = 0.028)	ADL SMM/ht^2^ No Cri-SMI Yes
Hicks	2005	Total body fat as confounder	B (SE, *p*-value) per unit higher baseline muscle mass CSA_trunk_ −0.014 (SE 0.006, NS) CSA_thigh_ 0.004 (SE 0.001, < 0.0001)	Performance CSA_trunk_ No CSA_thigh_ Yes
Meskers	2019	Body weight (kg) and fat mass (kg) as confounders for SMM and SMM/ht^2^ Body weight (kg) as confounder for SMM/wt*100%	B (95% CI) per unit higher baseline muscle mass ADL Females: SMM (kg) 0.00 (−0.07–0.07), SMM/wt*100% −0.01 (−0.07–0.04), SMM/ht^2^ 0.05 (−0.17–0.27) Males: SMM (kg) 0.04 (−0.01–0.08), SMM/wt*100% 0.06 (0.01–0.11), SMM/ht^2^ 0.17 (0.00–0.35) IADL Females: SMM (kg) 0.07 (−0.05–0.20), SMM/wt*100% 0.07 (−0.03–0.17), SMM/ht^2^ 0.22 (−0.17–0.619) Males: SMM (kg) 0.05 (−0.03–0.12), SMM/wt*100% 0.03 (−0.06–0.11), SMM/ht^2^ 0.21 (−0.11–0.52)	ADL SMM females no, males no SMM/wt*100% females yes, males no SMM/ht^2^ females no, males yes IADL SMM females no, males no SMM/wt*100% females no, males no SMM/ht^2^ females no, males no
Ohtsubo	2023	BMI as confounder	B (95% CI) per unit higher baseline muscle mass FIM-M: Low ALM/ht^2^ 0.022 (−0.06 to 0.113); Low PhA −0.109 (−0.194 to −0.024) SPPB: Low ALM/ht^2^ 0.013 (−0.074 to 0.100), Low PhA −0.108 (−0.190 to −0.026)	ADL ALM/ht^2^ no, PhA yes Performance ALM/ht^2^ no, PhA yes
Trombetti	2016	None (but association was reported to remain statistically significant after additional adjustment for confounders, including BMI)	B (95% CI) per unit higher baseline muscle mass 0.102 (0.002–0.201)	ADL CSA_thigh_ yes

B regression coefficient, CI confidence interval, ADL activities of daily living, IADL instrumental ADL

For muscle mass parameter abbreviations, see Table 2. For further study details, see Annex 3

Eleven prospective studies investigated the relationship between change in muscle mass and change in physical functioning. The main results of these 11 studies are provided in Table 5 (further details of these studies can be found in Annex 3). Nine out of 11 studies showed at least one statistically significant association indicating that greater loss of muscle mass was associated with greater decline in physical functioning. However, the association was not always consistently observed for a) all muscle mass parameters used in a single study [35, 51], b) men and women [54, 74], and c) the different physical function outcomes used in a single study [57].

**Table 5:** Overview of the 11 studies investigating the association between change in muscle mass over time and change in physical functioning in older adults

First author	Publication year	Body size adjustment	Most adjusted effect size	Statistically significant association (greater muscle loss is associated with greater decline in physical function)
Beavers	2013	None	B (SE, *p*-value): LM 0.006 (0.0034, 0.08), ALM 0.0061 (0.0033, 0.07), legLM 0.0043 (0.0033, 0.19), thighCSA 0.0084 (0.0035, 0.02)	LM, ALM, leg LM no Thigh CSA yes
Björkman	2012	Percent change in body weight as confounder	OR (95% CI) lowest tertile 7.74 (2.08–28.85), middle tertile 1.63 (0.48–5.52) versus highest tertile	SMM/ht^2^ yes
Duchowny	2020	Using ratios	Correlation coefficient (*p*-value) LM −0.11 (0.51), ALM −0.09 (0.57), ALM/ht^2^ −0.08 (0.60), ALM/weight −0.10 (0.56), D3 Cr muscle mass 0.29 (0.07), D3 Cr muscle mass/weight 0.33 (0.04)	LM, ALM, ALM/ht^2^, ALM/weight, D3 Cr muscle mass no D3 Cr muscle mass/weight yes
Fantin	2007	None	OR (95% CI) FFM 2.15 (1.10–4.20), leg FFM 2.53 (1.21–5.30)	FFM and leg FFM yes
Haight	2005	Using ratio	F: a one-unit gain in LM/FM reduced ADL onset by 65.5% (95% CI 21.8, 87.4). Not in M	L/FM females yes, LM/FM males No
Hirani	2017	Non-obese sample	OR (95% CI) ADL 1.30 (0.84–1.99), IADL 1.36 (1.05–1.76)	ALM with ADL no ALM with IADL yes
Osawa	2019	Baseline fat mass as confounder	B (SE, *p*-value) F 0.0035 (0.003, 0.17), M 0.007 (0.002, 0.01)	ALM females No, ALM males yes
Santanasto	2019	Baseline BMI + body weight change as confounders	OR (95% CI) LM 0.63 (0.43–0.91), ALM 0.56 (0.39–0.81), CSA tibia 0.61 (0.46–0.83)	LM, ALM, CSA tibia Yes
Scott	2020	Incorporated into contrast	*B* (95% CI) −0.031 (−0.082 to 0.019)	ALM no
Souza	2024	Waist circumference as confounder	*B* (95% CI) *M* −0.002 (−0.01–0.01), *F* −0.005 (−0.01–0.001)	SMM/ht^2^ no
Zuliani	2001	None	OR (95%) 5.60 (1.03–30.11)	BCM yes

M males, F females, B regression coefficient, SE standard error, CI confidence interval, OR odds ratio, ADL activities of daily living, IADL instrumental ADL

For muscle mass parameter abbreviations, see Table 2. For further study details, see Annex 3

Finally, eight studies investigated the association between baseline muscle mass and change in physical functioning, but did not provide the actual effect size [34, 88, 95], did not use regression analysis to test the association but rather Student’s t-test [69], Classification And Regression Tree (CART) analysis [93] or factor analysis [94], and/or adjusted for muscle strength, physical performance or sarcopenia status which are likely mediating the association between low muscle mass and decline in physical functioning [63, 64, 94] (Annex 8). Four of the eight studies found no association between lower muscle mass and change in physical functioning [34, 69, 88, 95], one study observed an association for D_3_CrMM/weight only (and not for D_3_CrMM or DXA parameters) [93], and for three studies no conclusion could be drawn [63, 64, 94].

## Discussion

The aim of this systematic review and meta-analysis was to provide a detailed overview of the current literature on the relationship between baseline muscle mass and prospective change in physical functioning in older men and women. The majority of studies contrasted low muscle mass with not-low muscle mass. Overall, the meta-analysis showed that a low muscle mass increased the risk of subsequent functional decline by 23%. Similarly, per SD higher muscle mass, the risk of functional decline was reduced by 11%. Results were similar for men and women. All six studies investigating the association between baseline muscle mass and change in physical functioning using a continuous outcome measure showed at least one statistically significant association. Finally, 9 out of 11 studies investigating change in muscle mass with change in physical functioning reported at least one statistically significant association. Based on this best available evidence, it can be concluded that lower muscle mass is a risk factor for functional decline in older adults.

This study also included stratified meta-analyses to potentially explain the high heterogeneity in the effect sizes observed in our previous meta-analysis [4]. The analyses showed a clear impact of the used body composition methodology to assess muscle mass, the used method to adjust for body size, and the type of outcome. The use of lower accuracy body composition methods, the lack of proper adjustment for body height and/or body fat, and the use of self-reported function outcomes may explain some of the earlier reported non-significant associations between low muscle mass and change in physical functioning.

This systematic review highlights the wide variety of body composition methods used to estimate muscle mass. Studies using methods that are generally considered more accurate, such as CT, DXA and D_3_Cr, showed strong and statistically significant associations between muscle mass and change in physical functioning. Overall, studies using more generally feasible, but less accurate, methods such as bioelectrical impedance and anthropometry showed no statistically significant association. Body circumferences of the arm or calf may not well reflect total body skeletal muscle mass, especially when BMI is not considered [105], and predicted muscle mass from BIA or anthropometric measures can be biased due to substantial individual prediction errors [106, 107], contributing to an overall underestimation of the true association.

The stratified meta-analyses suggested an influence of how researchers dealt with body size when investigating the association between muscle mass and subsequent change in physical functioning. When body size was ignored, or when only adjustment was made for body height, no overall statistically significant associations were observed. However, the overall association was significant when height and fat mass were included in the regression models as confounders. Of interest, the strongest associations were observed when the ratios of muscle mass over BMI, body weight, or fat mass were used. This is not surprising as multiple studies have shown that these denominators themselves are strong determinants of physical functioning in older adults [4, 108, 109] and thus will largely drive and inflate the association between these muscle mass ratios and low physical functioning. However, in the field of sarcopenia, it is of interest to determine whether muscle mass itself is too low and is negatively affecting physical functioning (and thus would require treatment). This conclusion would be difficult to draw when using these types of ratios. Indeed, the between-person variations in fat mass, body weight, and BMI are generally greater than the between-person variation in muscle mass, even in persons of similar height, making it difficult the isolate the role of muscle mass alone.

An interesting observation was that the high heterogeneity in the association between low muscle mass and change in physical functioning was reduced to 0% when selecting studies that used change in physical performance (i.e., decline in gait speed) as the study outcome. Moreover, the overall effect size was largest for studies using this objective outcome. Performance-based measures of physical functioning may be more sensitive to functional decline compared to self-reported measures [110]. A higher level of heterogeneity was observed for the associations of low muscle mass with self-reported mobility and (I)ADL limitations or disability, which could have been partly caused by the wide range in methods used to assess these outcomes, ranging from standard questionnaires (such as the Barthel Index) to data from long-term care indication centers.

In the 72 studies included in this review, 34 different muscle mass parameters were used. Some of these differences were due to the used body composition methodology, such as muscle cross-sectional area from CT or MRI scans, but others were due to researchers’ selection of the included body part (e.g., whole body, appendicular or leg lean soft tissue mass) and ways to express muscle mass (e.g., division by height squared, body weight, BMI or fat mass). Lastly, researchers used different criteria to define low muscle mass, even within the same racial/ethnicity group. These observations, together with the results of the stratified meta-analyses of the present study, clearly highlight the need for a global consensus on how low muscle mass in older adults should be operationalized.

To our knowledge, this review provides the most up-to-date evidence on the association between muscle mass and change in physical functioning. Further strengths include its focus on prospective studies to reduce reverse causation (i.e., poor physical functioning causing low muscle mass), its rigorous protocol to select the studies, and the inclusion of studies from geographically diverse regions, enhancing the generalizability of its findings. Final strengths are the conduct of a series of stratified meta-analyses to identify potential factors that influence the association between muscle mass and change in physical functioning, a detailed risk of bias assessment, and no suspicion of publication bias.

Some limitations of this review should be addressed. As described in “Results”, our meta-analyses involved heterogeneous definitions and categorizations of low muscle mass, making it impossible to stratify the analyses according to a definition or categorization. This may have contributed to the heterogeneity of the associations observed for low muscle mass. For example, quintile comparisons (lowest 20% versus highest 20%) typically capture a larger contrast and are likely to produce stronger effect sizes compared to binary classifications (lowest 20% versus highest 80%). Furthermore, after stratification, the meta-analyses often showed considerable statistical heterogeneity, which can be (partly) attributed to variation in sample characteristics, study design (including follow-up time), and confounders adjusted for in the statistical models. Another limitation would be the exclusion of studies conducted in specific patient groups, in order to reduce potential heterogeneity in the association between muscle mass and physical functioning caused by disease. Future studies are required to confirm our findings in patient groups. Finally, as this review includes a low number of studies performed in the hospital and nursing home setting, its generalisability to these settings is limited.

In conclusion, this extensive systematic review and meta-analyses show that lower muscle mass in older adults is associated with a greater risk of subsequent decline in physical functioning. Some of the non-significant associations reported in previous studies may have been influenced using a less accurate body composition method to estimate muscle mass, no or insufficient adjustment for body size, and self-reported measures of physical functioning as the study outcome.

## Supplementary Information

Below is the link to the electronic supplementary material.

Supplementary file1 (PDF 1136 KB)

## Data Availability

This study is based on published data, that are therefore already available for everyone.
