# Supplementary material for: The association between muscle mass and change in physical functioning in older adults: a systematic review and meta-analysis of prospective studies
Source: Eur Geriatr Med. 2025 May 23;16(5):1731–48. doi: 10.1007/s41999-025-01230-y (PMC12528211; doi:10.1007/s41999-025-01230-y)
Supplement: Supplementary file 1 — Supplementary file1 (PDF 1136 KB) [file 41999_2025_1230_MOESM1_ESM.pdf]

ANNEX 1. Search strategies

PubMed (7525)

Search	Query	Results
#7	Search: **#6 NOT ("Animals"[Mesh] NOT "Humans"[Mesh])**	7,525
#6	Search: **#5 NOT ("Review"[Publication Type] OR "Systematic Review" [Publication Type] OR "Meta-Analysis"[Publication Type] OR "Meta-Analysis as Topic"[Mesh] OR "meta-analysis"[tiab] OR "systematic review*"[tiab] OR "systematic literature review*"[tiab] OR "Letter"[Publication Type] OR "Editorial"[Publication Type] OR "Comment"[Publication Type])**	7,566
#5	Search: **#1 AND #2 AND #3 AND #4**	8,555
#4	Search: **"Epidemiologic Studies"[Mesh] OR "Observational Study" [Publication Type] OR "Case Reports" [Publication Type] OR cohort[tiab] OR (case[tiab] AND (series[tiab] OR control[tiab] OR report*[tiab] OR controll*[tiab] OR comparison[tiab] OR referent[tiab])) OR longitudinal[tiab] OR risk[tiab] OR "odds ratio"[tiab] OR etiol*[tiab] OR aetiol*[tiab] OR followup[tiab] OR "follow up"[tiab] OR "Prospective stud*"[tiab]**	9,172,087
#3	Search: **"Aged"[Mesh] OR "Geriatrics"[Mesh] OR "community dwel*"[tiab] OR elder*[tiab] OR geriatri*[tiab] OR aged[tiab] OR "older adult*"[tiab] OR senior*[tiab] OR senium*[tiab] OR septuagenarian*[tiab] OR octagenarian*[tiab] OR octogenarian*[tiab] OR nonagenarian*[tiab] OR centarian*[tiab] OR centenarian*[tiab] OR supercentenarian*[tiab] OR "advanced age"[tiab] OR ((older*[tiab] OR old[tiab] OR elder*[tiab] OR senior*[tiab]) AND (people*[tiab] OR subject*[tiab] OR patient*[tiab] OR age[tiab] OR adult*[tiab] OR man[tiab] OR men[tiab] OR male*[tiab] OR woman*[tiab] OR women*[tiab] OR female*[tiab] OR population*[tiab] OR person*[tiab]))**	5,296,498
#2	Search: **"Quality of Life"[Mesh] OR "Activities of Daily Living"[Mesh] OR "Disability Evaluation"[Mesh] OR "quality of life"[tiab] OR "life qualit*"[tiab] OR "activities of daily living"[tiab] OR "activity of daily living"[tiab] OR "activities of daily life"[tiab] OR "activity of daily life"[tiab] OR "daily living activit*"[tiab] OR "daily life activit*"[tiab] OR "adl"[tiab] OR "chronic limitation of activit*"[tiab] OR "qol"[tiab] OR "hrql"[tiab] OR "hrqol"[tiab] OR "disability evaluat*"[tiab] OR "mobility limit*"[tiab] OR "physical impair*"[tiab] OR "physically**	929,547

Search	Query	Results
	**impair*"[tiab] OR "ADL"[tiab] OR "IADL"[tiab] OR "walking disabilit*"[tiab] OR "functional limit*"[tiab] OR "functionally limit*"[tiab] OR "Barthel Ind*"[tiab] OR "Katz ind*"[tiab] OR "functional independence*"[tiab] OR "functional assessment*"[tiab] OR "SF-12"[tiab] OR "SF-36"[tiab] OR "physical function*"[tiab] OR "disabilit*"[tiab] OR "functional capacit*"[tiab] OR "physical assess*"[tiab] OR "physically assess*"[tiab] OR "functional status*"[tiab]**	
#1	Search: **"Muscle, Skeletal"[Mesh] OR "Muscular Atrophy"[Mesh] OR "Body Composition"[Mesh:NoExp] OR ((Mass*[tiab] OR Volume*[tiab] OR Thickness*[tiab] OR "Crosssectional area*"[tiab] OR "Cross sectional area*"[tiab] OR Circumference*[tiab] OR Densit*[tiab]) AND (muscle*[tiab] OR muscular[tiab] OR "body composition*"[tiab])) OR "lean mass"[tiab] OR "fat free mass"[tiab] OR "Sarcopenia*"[tiab]**	492,976

Embase (9671)

Search	Query	Results
#7	#6 NOT ([animals]/lim NOT [humans]/lim)	9671
#6	#5 NOT ('review'/it OR 'systematic review'/exp OR 'meta analysis'/exp OR (‘meta-analys*’ OR ‘systematic review*’ OR ‘systematic literature review*’):ti,ab,kw OR 'conference abstract'/it OR 'conference review'/it OR 'editorial'/it OR 'erratum'/it OR 'letter'/it OR 'note'/it OR 'short survey'/it OR ‘conference paper’/it OR ‘chapter’/it OR ‘tombstone’/it)	9763
#5	**#1 AND #2 AND #3 AND #4**	15,886
#4	'epidemiology'/exp OR 'observational study'/exp OR 'case report'/exp OR cohort OR ((case):ti,ab,kw AND (series OR control OR report* OR controll* OR comparison OR referent):ti,ab,kw) OR (longitudinal OR risk OR ‘odds ratio’ OR etiol* OR aetiol* OR followup OR ‘follow up’ OR ‘Prospective stud*’):ti,ab,kw	13,549,969

Search	Query	Results
#3	'aged'/exp OR 'geriatrics'/de OR (‘community dwel*’ OR elder* OR geriatri* OR aged OR ‘older adult*’ OR senior* OR senium* OR septuagenarian* OR octagenarian* OR octogenarian* OR nonagenarian* OR centarian* OR centenarian* OR supercentenarian* OR ‘advanced age’):ti,ab,kw OR ((older* OR old OR elder* OR senior*):ti,ab,kw AND (people* OR subject* OR patient* OR age OR adult* OR man OR men OR male* OR woman* OR women* OR female* OR population* OR person*):ti,ab,kw)	6,574,749
#2	'quality of life'/exp OR 'daily life activity'/exp OR 'ADL disability'/exp OR 'activity of daily living assessment'/exp OR 'functional status'/exp OR 'physical mobility'/exp OR 'disability assessment'/exp OR (‘quality of life’ OR ‘life qualit*’ OR ‘activities of daily living’ OR ‘activity of daily living’ OR ‘activities of daily life’ OR ‘activity of daily life’ OR ‘daily living activit*’ OR ‘daily life activit*’ OR ‘adl’ OR ‘chronic limitation of activit*’ OR ‘qol’ OR ‘hrql’ OR ‘hrqol’ OR ‘disability evaluat*’ OR ‘mobility limit*’ OR ‘physical impair*’ OR ‘physically impair*’ OR ‘ADL’ OR ‘IADL’ OR ‘walking disabilit*’ OR ‘functional limit*’ OR ‘functionally limit*’ OR ‘Barthel Ind*’ OR ‘Katz ind*’ OR ‘functional independence*’ OR ‘functional assessment*’ OR ‘SF-12’ OR ‘SF-36’ OR ‘physical function*’ OR ‘disabilit*’ OR ‘functional capacit*’ OR ‘physical assess*’ OR ‘physically assess*’ OR ‘functional status*’):ti,ab,kw	1,489,526
#1	'skeletal muscle'/exp OR 'muscle atrophy'/exp OR 'body composition'/de OR ((Mass* OR Volume* OR Thickness* OR ‘Crosssectional area*’ OR ‘Cross sectional area*’ OR Circumference* OR Densit*):ti,ab,kw AND (muscle* OR muscular OR ‘body composition*’):ti,ab,kw) OR (‘lean mass’ OR ‘fat free mass’ OR ‘Sarcopenia*’):ti,ab,kw	715,771

Web of Science (6669)

Search	Query	Results
#6	#4 AND #3 AND #2 AND #1 and Article or Early Access or Reprint (Document Types)	6,669
#5	#1 AND #2 AND #3 AND #4	7,960

Search	Query	Results
#4	TS=(cohort OR (case AND (series OR control OR report* OR controll* OR comparison OR referent)) OR longitudinal OR risk OR "odds ratio" OR etiol* OR aetiol* OR followup OR “follow up” OR “Prospective stud*”)	8,889,767
#3	TS=(“community dwel*” OR elder* OR geriatri* OR aged OR “older adult*” OR senior* OR senium* OR septuagenarian* OR octagenarian* OR octogenarian* OR nonagenarian* OR centarian* OR centenarian* OR supercentenarian* OR “advanced age” OR ((older* OR old OR elder* OR senior*) AND (people* OR subject* OR patient* OR age OR adult* OR man OR men OR male* OR woman* OR women* OR female* OR population* OR person*)))	5,823,612
#2	TS=("quality of life" OR “life qualit*” OR “living qualit*” OR "quality of living" OR "activities of daily living" OR "activity of daily living" OR "activities of daily life" OR "activity of daily life" OR “daily living activit*” OR “daily life activit*” OR "adl" OR "chronic limitation of activit*" OR "qol" OR "hrql" OR "hrqol" OR “disability evaluat*” OR "mobility limit*" OR "physical impair*" OR "physically impair*" OR "ADL" OR "IADL" OR "walking disabilit*" OR "functional limit*" OR "functionally limit*" OR "Barthel Ind*" OR "Katz ind*" OR "functional independence*" OR "functional assessment*" OR "SF-12" OR "SF-36" OR "physical function*" OR "disabilit*" OR "functional capacit*" OR "physical assess*" OR "physically assess*" OR "functional status*")	1,073,197
#1	TS=(((Mass* OR Volume* OR Thickness* OR “Crosssectional area*” OR “Cross sectional area*” OR Circumference* OR Densit*) AND (muscle* OR muscular OR “body composition*”)) OR “lean mass” OR “fat free mass” OR "Sarcopenia*")	273,844

ANNEX 2. Risk of Bias assessment criteria

Criterium 1: Was selection of exposed and non-exposed cohorts drawn from the same population?

- Definitely yes: if the study selected participants from the same cohort study;
- Probably yes: if the study uses data from multiple cohorts in the analysis;
- (Probably/definitely no: not applicable due to exclusion criteria in the screening phase).

Criterium 2: Can we be confident in the assessment of exposure (muscle mass)?

- Definitely yes: description of assessment needs to be complete. DXA and CT measurement descriptions should at least include information about type of machine, manufacturer and software used. BIA description should include information about the protocol, type of machine, manufacturer, prediction equation and frequency;
- Probably yes: if most but not all information is present;
- Probably no: not clearly reported;
- Definitely no: not reported.

Criterium 3: Can we be confident that the outcome of interest (decline in physical functioning) was not present at the start of the study?

- Definitely yes: if reported clearly that decline of physical functioning was not present at the start of the study;
- Probably yes: if reported, but could be more explicit;
- Probably/definitely no: not clearly reported;
- Definitely no: not reported.

Criterium 4: Did the study match exposed and unexposed for all variables that are associated with the outcome of interest (physical functioning) or did the statistical analysis adjust for these prognostic variables?

Matching did not occur in the selected papers, therefore we focused on the adjustment for confounding variables. The use of muscle mass ratios (e.g., muscle mass/weight, or muscle mass/BMI etc) is not considered as adjustment.

- Definitely yes: the study adjusted for all key confounders, including demographic factors, lifestyle variables, and other relevant variables, such as mental and cognitive functioning. If appropriate, baseline physical functioning was also adjusted for;
- Probably yes: the study adjusted for at least demographic factors, disease variables, and lifestyle variables, and baseline physical functioning if appropriate, but did not adjust for some other relevant variables (e.g. mental or cognitive functioning);
- Probably no: the study adjusted for at least demographic factors and disease variables, but other key confounders are missing;
- Definitely no: the study did not adjust for any of the key confounders.

Criterium 5: Can we be confident in the assessment of the presence or absence of prognostic factors (confounding variables)?

- Definitely yes: valid assessment of confounding variables, thoroughly reported;
- Probably yes: valid assessment of confounding variables, less thoroughly reported.
- Probably no: validity of assessment can be questioned (e.g. self-reported weight)
- Definitely no: not reported.

Criterium 6: Can we be confident in the assessment of outcome (physical functioning)?
Risk of Bias assessment depends on the type of outcome assessment. In some studies self-created outcomes such as worsening of functional limitations are being used. In that case we assess the type of assessment AND the definition of worsening of functional limitations.

- Definitely yes: if outcome was measured by validated questionnaire or objective tests, and thoroughly reported;
- Probably yes: valid assessment, but not thoroughly reported;
- Probably no: unvalidated set of questions used, or not clearly reported;
- Definitely no: not reported.

Criterium 7: Was the follow-up of cohorts adequate?

- Definitely yes: if follow-up and loss to follow-up is clearly reported;
- Probably yes: follow-up and loss to follow-up numbers are present, but not clearly reported;
- Probably no: missing information about either follow-up or loss to follow-up;
- Definitely no: follow-up and loss to follow-up not reported.

Criterium 8: Are there any (other) flaws in this study?
These may concern flaws in study design, statistical analyses or conclusions drawn from the results. If there is a flaw, motivation should be given.

- No: no (other) flaws present;
- Yes: yes, a flaw is present
    - Motivation: ….

ANNEX 3. General characteristics, muscle mass parameters[1] and change in physical functioning outcomes[1] of the 72 included studies

First author	Publication year	Reference	Country of study population	Setting	No. of females and males	Follow-up time physical functioning (mos)	Muscle mass assessment method	Muscle mass parameter	How was muscle mass parameter at baseline used in model	Physical functioning outcome(s)[2]
Abay	2022	27	USA	community	802 F, 909 M	6	DXA	ALM/BMI	Low (M<0.789, F<0.512) vs not low (101), and per SD lower	ADL: any new inability or help needed to bathe, dress, or transfer.
Aliberti	2020	28	Brazil	hospital	421 F, 244 M	12	ANTHR	CC	Low (M≤34 cm, F≤33 cm) vs not low (107)	ADL: any new need for help in eating, transferring, dressing, toileting, and bathing. Mobility: any worsening ability to walk across the room.
Amigues	2013	29	France	community	975 F	48	DXA	ALM/ht2	Lowest (<5.82) vs highest quartile, and per SD lower	IADL: any new self-reported loss of performing eight IADL items without assistance.
Andrews	2022	30	USA	community	742 F, 806 M	16	DXA	ALM/BMI	Low (M<0.789, F<0.512) (101), and per SD lower	ADL: any new inability or help needed to perform bathing, dressing, or transferring in and out of chairs.
Auyeung	2013	31	China	community	1587 F, 1566 M	48	DXA	FM/legLM, FM/FFM, Wt/FFM	Per SD higher	MOB: any new or worsening physical functioning in moderate activities (such as moving a table, pushing a vacuum cleaner) or climbing several flights of stairs.
Baker	2018	32	USA	community	1460 F, 1386 M	NR	DXA	ALM/BMI, $ALM/ht^2/FM/ht^2$ Z score, $ALM/ht^2/FM/ht^2$	Low ALM/BMI unclear from paper. Low defined as $ALM/ht^2/FM/ht^2$ Z score≤ -1. Per SD higher Z score (103)	ADL: any new self-reported disability, defined as severe difficulty or inability to in walk 1/4 mile and/or

										climb 10 steps, needing equipment to ambulate, or having any difficulty performing activities of daily living (i.e., getting in and out of bed or chairs, bathing or showering, and dressing).
Baker	2020	33	USA	community	2843 FM	NR	DXA	ALM/ht^2, ALM/ht^2/FM/ht^2 Z score	Low (M<7.26, F<5.45) vs not low, in non-obese only. Low defined as ALM/ht2/FM/ht2 Z score≤ -1, in non-obese only (105)	ADL: any new self-reported disability, defined as severe difficulty or inability to walk 1/4 mile and/or climb 10 steps, needing equipment to ambulate, or having any difficulty performing activities of daily living (i.e., getting in and out of bed or chairs, bathing or showering, and dressing).
Baumgartner	2004	34	USA	community	451 FM	96	DXA	ALM/ht^2	Low (M<7.26, F<5.45) vs not low (105)	IADL: no longer being able to do two or more tasks without help: using the telephone, accessing transportation, getting groceries, making meals, doing housework, doing handyman work, doing laundry, taking medications, and managing money.
Beavers	2013	35	USA	community	1158 F, 1148 M	48	DXA, CT	LM, ALM, LegLM, CSA_{thigh}	Per SD higher. Also: per SD change over time	Performance: change in 20 m walking speed (cont)

Björkman	2019	36	Finland	community	194 F, 68 M	48	BIA (BIS)	SMM/ht^2, calf intracellular resistance skeletal muscle index	Per SD higher	ADL: RAND-36 questionnaire score (cont)
Björkman	2012	37	Finland	nursing home	83 FM	6	BIS	SMM/ht^2	Tertiles of change over time	ADL: change in total MDS 2.0 long score (cont). Mobility: change in sum of three MDS items (transfer between surfaces, walking in room, and toilet use) (cont).
Broadwin	2001	38	USA	community	634 F, 417 M	48	BIA	FFM/wt*100%	Lowest (M<75.7, F<67.1) vs highest quintile	ADL: any new disability in nine tasks representing upper and lower body function and mobility. Mobility: any new disability walking 2–3 blocks and climbing up 10 stairs.
Buchman	2021	39	USA	community	1107 F, 359 M	67	BIA	SMM	Per SD higher	IADL: any new inability to do activities without help based on telephone use, meal preparation, money management, medication management, light and heavy housekeeping, shopping, and local travel. ADL: any new inability to do activities without help based on feeding, bathing, dressing, toileting, transferring, and walking across a small room. Mobility: any new inability to do

										activities without help based on Rosow-Breslau scale (walking up and down a flight of stairs, walking a half mile, and doing heavy housework like washing windows, walls, or floors).
Cawthon	2015	40	USA	community	3726 M	55	DXA	ALM/ht², ALM residual regressed on height and FM	Low (M≤7.23) vs not low (105). Low defined as residual <-0.204 (106)	ADL: any new inability to walk 2–3 blocks, climb 10 steps without resting, prepare meals, shop, or do heavy housework.
Cawthon	2020	41	USA, Sweden, Hong Kong, Australia	community	1500 F, 6505 M	82	DXA	ALM, ALM/ht²	Low (ALM M<19.75, F<15.02 (101), ALM/Ht² M<7.26, F<5.45) vs not low (105)	Mobility: new self-reported mobility limitation (harmonized across cohorts)
Cawthon	2021a	42	USA	community	1425 M	26	D3Cr, DXA	MM/wt, ALM/ht², ALM/BMI, ALM/wt, ALM	Lowest quartile vs highest quartile (cut-points not provided) and per SD lower	IADL: new disability in at least one task (preparing meals, doing heavy housework, shopping for groceries/clothes, managing money, managing medications, driving). Mobility: new disability in at least one task (walking two to three blocks on level ground, climbing 10 steps without resting, and carrying or lifting 10 pounds).
Cawthon	2021b	43	USA	community	5836 M	168	DXA	ALM/ht²	Highest quintile versus lowest (<7.21)	Mobility: any new difficulty in walking 2–3 blocks or climbing 10 stairs.

Cawthon	2019	44	USA	community	1062 M	42	D3Cr, DXA	MM/wt, ALM/ht^2, MM/ht^2, ALM/wt, ALM/BMI	Lowest quartile (ALM/ht2 <6.9, MM/wt <0.28) versus highest, and per SD higher. Quartile cut-points for other muscle mass not provided.	Mobility: any new self-reported difficulty walking 2–3 blocks or climbing 10 steps.
Cesari	2015	45	Italy	community	526 F, 396 M	109	CT	CSA_{tibia}	Per SD higher residuals of regression on height, and of regression on height and fat mass.	ADL: any new loss of autonomy in bathing, dressing, toileting, transfer, continence, and feeding.
Chiba	2021	46	Japan	community	1197 F, 952 M	60	BIA	ALM/ht^2	Low (M<7.0, F<5.7) vs not low (99)	ADL: disability was defined as new requirement of long-term care according to LTCI certification.
Chiles Shaffer	2017	47	USA	community	180 F, 191 M	60	DXA	ALM, ALM/BMI	Low (ALM M<21.38, F<14.12, ALM/BMI M<0.735, F <0.591) vs not low. Cut-off values based on CART analyses in own sample.	Performance: incidence of 6 m slow walking speed (<0.80 m/s)
Costanzo	2020	48	Italy	community	198 F, 171 M	36	BIA	ALM/ht^2	Low (M<7, F<6) vs not low, in those with normal strength (98)	Performance: Loss of ability to walk 400 m within 15 min without having to sit or needing help of another person or walker.
Davies	2022	49	Spain	community	1305 FM	36	DXA	ALM/BMI	Low (M<0.65, F<0.54) vs not low (101, adjusted for own sample)	ADL: new worsening of score on Katz index.
Delmonico	2007	50	USA	community	1543 F, 1433 M	60	DXA	ALM/ht^2, ALM residuals of regression on height and FM	Low (ALM/ht^2 M<7.25, F<5.67) based on lowest 20th percentile sample) vs not low.	Mobility: self-reported difficulty walking one-quarter of a mile or climbing 10 steps without resting at two consecutive 6-month intervals. Performance: change in Established Populations for the

										Epidemiologic Study of the Elderly (EPESE) Short Physical Performance Battery (5x chair stands, gait speed, standing balance).
Duchowny	2020	51	USA	community	40 M	19	DXA, D3Cr	LM, ALM, ALM/ht^2, ALM/wt, MM, MM/wt	Absolute change and percent change over time	Performance: percent change in 6 m walking speed (cont).
Fantin	2007	52	Italy	community	97 F, 62 M	66	DXA	AppFFM, FFM, LegFFM	Per unit higher (AppFFM). Also: change over time (FFM, AppFFM, LFFM)	ADL: any worsening in disability score based on bathing, getting out of bed, dressing, eating unaided, walking across a small room, walking 800 meters, climbing stairs, doing heavy housework, shopping, using the telephone, doing light housework, preparing meals.
Franzon	2019	53	Sweden	community	127 M	60	DXA	ALM/ht^2	Per SD higher	ADL: independence in personal ADL (bathing, dressing, toileting). Mobility: ability to walk outdoors alone assistive device allowed).
Haight	2005	54	USA	community	947 F, 708 M	77	BIA	LM/FM	Per unit increase over time	ADL: any new onset of decline, defined as reporting ‘a lot of difficulty’ doing one or more of the 10 activities or not doing at least one because being unable or advised by a physician not to do so.

Hicks	2005	55	USA	community	621 F, 573 M	36	CT	$CSA_{trunk\ L4\text{-}L5}$, CSA_{thigh}	Per unit higher	Performance: Health ABC physical performance battery (5x chair stands, standing balance, walk pace 6 m, walk pace narrow 6 m) (cont)
Hirani	2015	56	Australia	community	955 M	60	DXA	ALM	Low (M<19.75) vs not low (108)	ADL: new disability defined as needing help with one or more activities (walking across a small room, bathing, grooming, dressing, eating, transferring from a bed to a chair, and using the toilet).
Hirani	2017	57	Australia	community	917 M	60	DXA	ALM	Low (M<0.789) vs not low at all three timepoints (101). Data extracted from non-obese only.	ADL: disability at each timepoint was defined as needing help with one or more activities of the modified Katz scale (walking across a small room, bathing, grooming, dressing, eating, transferring from a bed to a chair, and using the toilet). IADL: disability at each timepoint was defined as needing help to perform ≥1 of the IADL tasks.
Ishii	2020	58	Japan	community	4838 F, 4391 M	24	DXA	ALM/ht^2	Low (M<7.0, F<5.7) vs not low (99)	ADL: disability was defined as new requirement of long-term care according to LTCI certification.

Jang	2018	59	Korea	community	741 F, 602 M	22	BIA	ALM/ht^2, ALM/wt, ALM/BMI	Lowest quintile (ALM/BMI M<0.693, F<0.466; ALM/ht^2 M<6.439, F<5.197; ALM/wt M<27.547, F<21.383) vs highest quintile	ADL: any new disability based on seven ADL activities of daily living (bathing, continence, dressing, eating, toileting, transferring, and washing face and hands). IADL: any new disability based on ten IADL activities (food preparation, household chores, going out a short distance, grooming, handling finances, laundry, managing own medications, shopping, transportation, and using a telephone).
Janssen	2006	60	USA	community	1964 F, 1730 M	96	BIA	SMM/ht^2	Low (M<7.0, F<5.7) vs not low (102)	ADL: any new difficulty performing six tasks (heavy housework, light housework, shopping, preparing meals, paying bills, and using the telephone).
Kitamura	2021	61	Japan	community	935 F, 916 M	70	BIA	ALM/ht^2	Low (M<7.0, F<5.7) vs not low, in those with no low grip and no low gait speed (100)	ADL: disability was defined as new requirement of long-term care according to LTCI certification.
Lam	2020	62	Hong Kong	community	2000 F, 2000 M	48	DXA	ALM/ht^2, ALM/wt, ALM/BMI	Per SD higher	ADL: any new occurrence or worsening of limitations based on two items (climbing stairs, and carrying out the following household activities, such as

										moving chairs or tables).
Legrand	2014	63	Belgium	population-based	267 F, 154 M	20	ANTHR	SMM	Per tertile higher	ADL: a decline in ADL of at least 3 points, based on six activities (climbing stairs, walking 5 minutes outdoors without resting, sitting down in and standing up from a chair, dressing and undressing oneself, using own or public transportation, and cutting one's own toenails).
Lera	2020	64	Chili	community	299 F, 131 M	58	DXA, ANTHR	LM/FM	Per unit higher	ADL: limitation in at least one activity of daily living, two instrumental activities of daily living, or three mobility limitations.
Masugi	2022	65	Japan	community	842 F, 681 M	24	BIA	ALM/ht^2	Per SD higher	IADL: new IADL dependency was defined as a score ≤4 based on five activities (use public transportation by yourself, shop for daily necessities, prepare meals, pay bills, handle own banking).
McLean	2014	66	USA, Italy	community	1581 F, 4034 M	36	DXA	ALM, ALM/BMI	Low (ALM M<19.75, F<15.02; ALM/BMI M<0.789, F<0.512) vs not low (101)	Performance: incident slow walking speed (≤0.8 m/s).
Meskers	2019	67	Netherlands	hospital	186 F, 192 M	3	BIA	SMM, SMM/wt*100%, SMM/ht^2	Per unit higher	ADL: change in Katz score, which ranges from 0 to 6 points, with negative change scores indicating an increase in

										dependency (cont). IADL: change in Lawton and Brody score, which ranges from 0 to 8 points, with negative change scores indicating an increase in dependency (cont)
Nagae	2022a (#139)	68	Japan	hospital	149 F, 107 M	NR	BIA, US	BATT, SMM/ht^2	Per unit higher	ADL: a ≥10% decrease in the Barthel Index score, based on 7 items (eating, grooming, toilet use, bathing, dressing, bowels, and bladder). Mobility: a ≥10% decrease in the Barthel Index score, based on 3 items (transfers, walking, and stairs).
Nagae	2022b (#142)	69	Japan	hospital	34 F, 59 M	3	US	BATT	Per unit higher	ADL: any decline in Barthel Index score, based on 10 items (feeding, transfers, grooming, toilet use, bathing, mobility, stairs, dressing, bowels, and bladder).
Oh	2023	70	Korea	community	1034 F, 925 M	24	DXA	ALM/ht^2	Low (M<7.0, F<5.4) vs not low (100)	IADL: new inability to perform 2 or more of the 10 IADL domains (decorating, housework, preparing meals, laundry, short outings, using transportation, shopping, handling money, using the telephone, and taking medicines).

Ohtsubo	2023	71	Japan	Hospital	320 F, 123 M	2	BIA	PhA, ALM/ht^2	Low (ALM/ht2 M<7.0, F<5.7 (99); PhA M<4.05, F<3.55) vs not low (109)	ADL: functional independence score for motor function (FIM-M) based on 13 items from the motor category, including self-care (cont). Performance: Short Physical Performance Battery score based on three tests (cont).
Orwoll	2020	72	USA	community	1065 M	42	D3Cr, DXA	MM/wt, ALM/ht^2	Lowest quartile (MM/wt M<0.28; ALM/ht^2 M<6.93) vs highest	Mobility: any new self-reported difficulty walking 2–3 blocks or climbing 10 steps.
Orwoll	2022	73	USA	community	832 M	26	D3Cr	MM	Per SD higher	Mobility: any new self-reported difficulty walking 2–3 blocks or climbing 10 steps.
Osawa	2019	74	USA	community	575 F, 539 M	48	DXA	ALM	Per unit increase over time	Performance: change in 400 m gait speed (cont).
Pérez-Zepeda	2016	75	Mexico	community	378 F, 367 M	24	ANTHR	CC	Lowest quartile (M<35, F<34) vs highest	Mobility: any new reported difficulty to walk one block or to climb one flight of stairs.
Reinders	2015	76	Iceland	community	1651 F, 1208 M	62	CT	CSA_{thigh}	Per sex-specific SD higher	Mobility: any new reported having much difficulty or unable to walk 500 m and/or climb 10 steps. Performance: a decline of ≥0.1 m/s in 6 m walking speed.
Rossi	2020	77	Italy	community	117 F, 67 M	66	DXA	ALM/ht^2	Lowest tertile (M<7.00, F<5.18) vs highest	ADL: any worsening in disability score based on bathing, getting out of bed, dressing, eating unaided, walking across

										a small room, walking 800 m, climbing stairs, doing heavy housework, shopping, using the telephone, doing light housework, preparing meals.
Santanasto	2019	78	Tobago	community	505 M	74	DXA, CT	LM, ALM, CSA_{tibia}	Per SD decrease over time	Mobility: any new difficulty walking 2-3 blocks or climbing up 10 steps due to a health/physical problem.
Scott	2020	79	Australia	community	1326 M	36	DXA	ALM	Change over time in four groups based on ALM and FM. Change over time in the residuals regressed on change in FM	Performance: change in 6 m walking speed (cont).
Seino	2022	80	Japan	community	903 F, 862 M	64	BIA	ALM/ht^2	Low (based on lowest sex-specific 10th percentile sample) vs reference value (M=7.0 and F=5.7).	ADL: disability was defined as the onset of long-term care needs at the support level 1 or above according to LTCI certification.
Shimada	2021	81	Japan	community	2299 F, 2262 M	49	BIA	ALM/ht^2	Low (M<7.0, F<5.7) vs not low (100)	ADL: new disability was defined as being certified by the LTCI as requiring support or care.
Souza	2024	82	UK	community	1382 F, 1298 M	96	ANTHR	SMM/ht^2	Lowest sex-specific 20th percentile of sample (F<6.52, M<9.24) at baseline, and after 4 and 8 years.	Performance: 2.4 m gait speed (cont) at baseline, and after 4 and 8 y
Tager	2004	83	USA	community	947 F, 707 M	60	BIA	LM/FM	Per 0.5 unit higher	ADL: at least 1 self-reported limitation (reporting a lot of difficulty doing one or more of the functions, or not doing at least

										one function because they were unable or were advised by a doctor not to do so) based on 10 domains of upper and lower body physical function.
Trombetti	2016	84	USA	community	24 F, 24 M	36	CT	CSA_{thigh}	Per unit higher	ADL: change in the physical component summary score of the 36-item Short Form Survey (cont).
Uemura	2020	85	Japan	community	2084 F, 2228 M	24	BIA	PhA, ALM/BMI	Per SD higher	ADL: incident disability was defined as new certification of LTCI service at any level.
Verlaan	2017	86	Netherlands	hospital	281 FM	3	BIA	SMM, SMM/ht^2, SMM/wt, FFM	Per unit higher	ADL: living independently, defined as living at home as opposed to assisted living in care home, nursing home or rehabilitation center.
Visser	1998	87	USA	community	1785 F, 1489 M	36	BIA	FFM	Lowest quintile (M<43.5, F<29.0) vs highest	Mobility: newly reported difficulty in walking 0.5 miles or walking up 10 steps.
Visser	2005	88	USA	community	1345 F, 1286 M	30	CT	CSA_{thigh}	Lowest quartile (white M<109, black M<110, white W<67, black W<71) vs highest	Mobility: two consecutive self-reports of any difficulty walking one-quarter mile or climbing 10 steps during follow-up.
Wagner	2022	89	France	community	823 M	96	DXA	LegLM, LegLM/Leg length^2	Per unit higher at baseline	Performance: no longer able to complete a test at follow-up (5x chair stands, static balance, dynamic balance, forward and backward tandem walk).

Woo	2001	90	Hong Kong	community	612 F, 559 M	36	ANTHR	FFM, CAMA	Highest vs lowest quartile, cut-points not provided	ADL: new dependency was defined as a Barthel Index score at follow-up <20. Performance: At follow-up, being in top quartile of time needed to walk a distance of 16 feet (>17.04 s).
Woo	2016	91	Hong Kong	community	1587 F, 1566 M	48	DXA	ALM, ALM/BMI	Low (ALM M<15.65, F <11.26; ALM/BMI M<0.6933, F<0.522) vs not low for ADL. Low (ALM M<15.61, F <12.42; ALM/BMI M<0.7223, F<0.4718) vs not low for performance. Cut-off values based on CART analyses in own sample.	ADL: new report of either a little or a lot of difficulty in climbing stairs or carrying out household activities such as moving chairs or tables. Performance: incident slow 6 m walking speed (<0.8 m/s).
Woo	2009	92	Hong Kong	community	1587 F, 1566 M	48	DXA	ALM/ht^2	Low (M<5.72, F <4.82) vs not low (104)	ADL: any new impairment in walking 2-3 blocks outdoors on level ground, climbing 10 steps without resting, preparing own meals, doing heavy housework like scrubbing floors or washing windows, and shopping for groceries or clothes.
Woo	2018	93	Hong Kong	community	1508 F, 1525 M	48	DXA	ALM/FM	Lowest quintile (M<1.04, F<0.609) vs highest	ADL: new report of either a little or a lot of difficulty in climbing stairs or carrying out household activities such as moving chairs or tables. Performance: incident slow 6 m

										walking speed (<0.8 m/s).
Zanker	2020	94	USA	community	1098 M	26	DXA, D3Cr	ALM/ht^2, ALM/wt, ALM/BMI, MM, MM/wt	Per unit higher	Mobility: new self-reported inability to perform 1 or more of the mobility measures (walking 2-3 blocks on level ground, climbing 10 steps without resting, and carrying or lifting 10 lbs.).
Zanker	2022	95	USA	community	1345 M	26	DXA, CT, D3Cr	MM/wt, CSA_{tibia}, ALM/wt, ALM/BMI	Per unit higher baseline	Performance: 6 m gait speed and 5x chair stands. ADL: reported inability for eating or feeding oneself, transferring, bathing or showering, and toileting. IADL: reported inability for preparing meals, doing heavy housework, shopping for groceries or clothes, managing money, managing medications, and driving. Mobility: reported inability for walking 2–3 blocks on level ground, climbing 10 steps without resting, and carrying or lifting 10 pounds.
Zhao	2024	96	China	community	4768 (ADL) and 4362 (IADL), about 50.8% M	36	ANTHR	ALM/ht^2	Lowest sex-specific 20th percentile of sample (F<5.07, M<6.87) vs not low	ADLs: new onset of difficulty to perform daily physical tasks, including dressing, bathing, feeding, transferring from bed to coach, using the toilet, and urinary and

										faecal continence. IADLs: new onset of having some difficulty living independently: making housework, cooking, shopping, managing money, and taking medication.
Zoico	2007	97	Italy	community	85 F, 60 M	24	DXA	ALM/ht^2	Below median (M<7.6, F <5.4) vs above median	ADL: any new difficulty in any item of the questionnaire, including an ADL scale, three Rosow Breslau physical function items and an IADL scale. Mobility: any new difficulty walking 800 m or climbing stairs.
Zuliani	2001	98	Italy	nursing home	79 F, 19 M	24	BIA	BCM	Decrease vs no decrease over time	ADL: any decline in Katz Index score.

[1] Used in the relevant statistical model(s) testing the association between muscle mass and change in physical functioning. [2] ADL=self-reported limitations or disability in activities of daily living, IADL=self-reported limitations or disability in instrumental ADL, Mobility=self-reported mobility limitations or disability, Performance=physical functioning based on one or more performance tests.

ALM = appendicular lean soft tissue mass, ANTHR = anthropometry, App = appendicular, BATT= bilateral anterior thigh, thickness, BCM = body cell mass, BIA = bioelectrical impedance, BIS = Bioelectrical impedance spectroscopy, BMI = body mass index, CAMA = corrected arm muscle area, CC = calf circumference, Cont=continuous variable, CSA = cross-sectional area, CT = computer tomography, D3Cr = D3-creatine dilution, DXA = dual-energy x-ray absorptiometry, F = females, FFM = fat-free mass, FM = total body fat mass, Ht = body height, LM = lean soft tissue mass, LTCI = long-term care insurance system, M = males, MM = muscle mass, NR = not reported, PhA = Phase angle, SD = standard deviation, SMM = whole body skeletal muscle mass, US = ultrasound, Wt = body weight, CART= Classification And Regression Tree.

ANNEX 4. Risk of bias evaluation of the 72 included studies

First author	Publication year	Reference	Criterium 1[1]	Criterium 2	Criterium 3	Criterium 4	Criterium 5	Criterium 6	Criterium 7	Risk of Bias category[2]
Abay	2022	27								L
Aliberti	2020	28								L
Amigues	2013	29								L
Andrews	2022	30								L
Auyeung	2013	31								H
Baker	2018	32								S
Baker	2020	33								S
Baumgartner	2004	34								L
Beavers	2013	35								S
Björkman	2019	36								S
Björkman	2012	37								S
Broadwin	2001	38								S
Buchman	2021	39								S
Cawthon	2015	40								L
Cawthon	2020	41								S
Cawthon	2021a	42								L
Cawthon	2021b	43								L
Cawthon	2019	44								L
Cesari	2015	45								L
Chiba	2021	46								L
Chiles Shaffer	2017	47								L
Costanzo	2020	48								L
Davies	2022	49								L
Delmonico	2007	50								L
Duchowny	2020	51					n/a			S
Fantin	2007	52								S
Franzon	2019	53								S
Haight	2005	54								S
Hicks	2005	55								L
Hirani	2015	56								L
Hirani	2017	57								S
Ishii	2020	58								L
Jang	2018	59								L
Janssen	2006	60								S
Kitamura	2021	61								S

Lam	2020	62								S
Legrand	2014	63								S
Lera	2020	64								H
Masugi	2022	65								S
McLean	2014	66								S
Meskers	2019	67								L
Nagae	2022a	68								L
Nagae	2022b	69					n/a			S
Oh	2023	70								S
Ohtsubo	2023	71								S
Orwoll	2020	72								L
Orwoll	2022	73								L
Osawa	2019	74								S
Pérez-Zepeda	2016	75								S
Reinders	2015	76								L
Rossi	2020	77								S
Santanasto	2019	78								S
Scott	2020	79								S
Seino	2022	80								S
Shimada	2021	81								S
Souza	2024	82								L
Tager	2004	83								L
Trombetti	2016	84								H[3]
Uemura	2020	85								L
Verlaan	2017	86								H
Visser	1998	87								L
Visser	2005	88								L
Wagner	2022	89								L
Woo	2001	90					n/a			S
Woo	2016	91								S
Woo	2009	92								S
Woo	2018	93								S
Zanker	2020	94								L
Zanker	2022	95								L
Zhao	2024	96								L
Zoico	2007	97								S
Zuliani	2001	98								S

[1] Criterium 1: Was selection of exposed and non-exposed cohorts drawn from the same population?; Criterium 2: Can we be confident in the assessment of exposure (muscle mass)?; Criterium 3: Can we be confident that the outcome of interest (decline in physical functioning) was not present at the start of the study?; Criterium 4: Did

the study match exposed and unexposed for all variables that are associated with the outcome of interest (physical functioning) or did the statistical analysis adjust for these prognostic variables?; Criterium 5: Can we be confident in the assessment of the presence or absence of prognostic factors (confounding variables)?; Criterium 6: Can we be confident in the assessment of outcome (physical functioning)?; Criterium 7: Was the follow-up of cohorts adequate? Dark green: definitely yes, light green: probably yes, yellow: probably no, red: definitely no. n/a: not applicable.

[2] L=low risk of bias, S=some risk of bias, H=high risk of bias.

[3] Criterium 8 (Are there any other flaws in the paper?) was answered with 'yes', due to the very small sample size (F=24, M=24). All other studies scored 'no' for this criterium.

Annex 5. Funnel plots of the two main random-effect meta-analyses

Funnel plot of random-effect meta-analysis 1: Risk of developing functional decline in older adults with low baseline muscle mass versus those with not-low muscle mass.

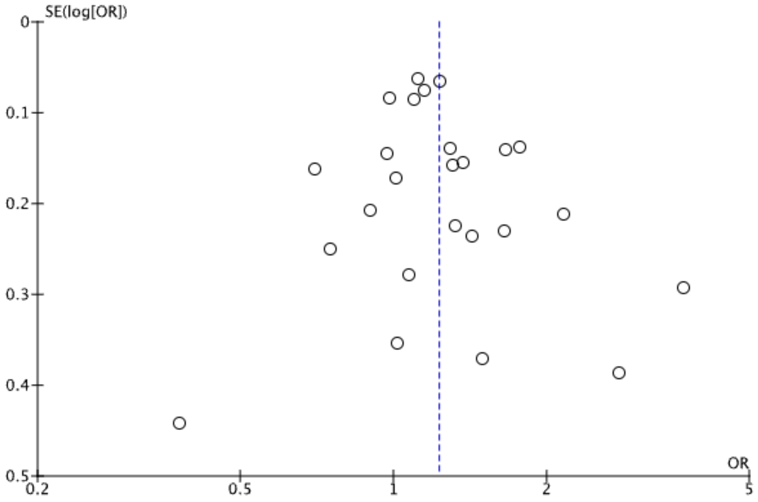

Funnel plot of random-effect meta-analysis 2: Risk of developing functional decline in older adults per standard deviation higher baseline muscle mass.

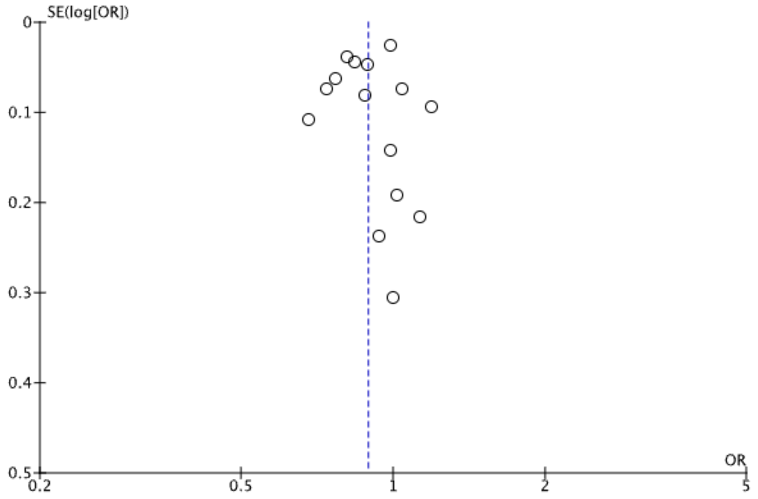

ANNEX 6. Random-effect meta-analysis of the risk of developing functional decline in older adults with low baseline muscle mass versus those with not-low muscle mass, categorized by a) type of outcome measure, b) accuracy of the body composition method to assess muscle mass, and c) adjustment for body size.

6a. Type of outcome measure: gait speed decline (n=5)

Study or Subgroup	log[Odds Ratio]	SE	Weight	Odds Ratio IV, Random, 95% CI
Chiles-Shaffer 2017	0.0198	0.3537	5.9%	1.02 [0.51, 2.04]
Costanzo 2020	0.3988	0.3711	5.3%	1.49 [0.72, 3.08]
McLean 2014	0.5068	0.1407	37.1%	1.66 [1.26, 2.19]
Woo 2001	0.3577	0.2362	13.2%	1.43 [0.90, 2.27]
Woo 2018	0.571	0.1382	38.5%	1.77 [1.35, 2.32]
**Total (95% CI)**			**100.0%**	**1.61 [1.36, 1.91]**

Heterogeneity: $Tau^2 = 0.00$; $Chi^2 = 2.48$, df = 4 (P = 0.65); $I^2 = 0\%$

Test for overall effect: Z = 5.57 (P < 0.00001)

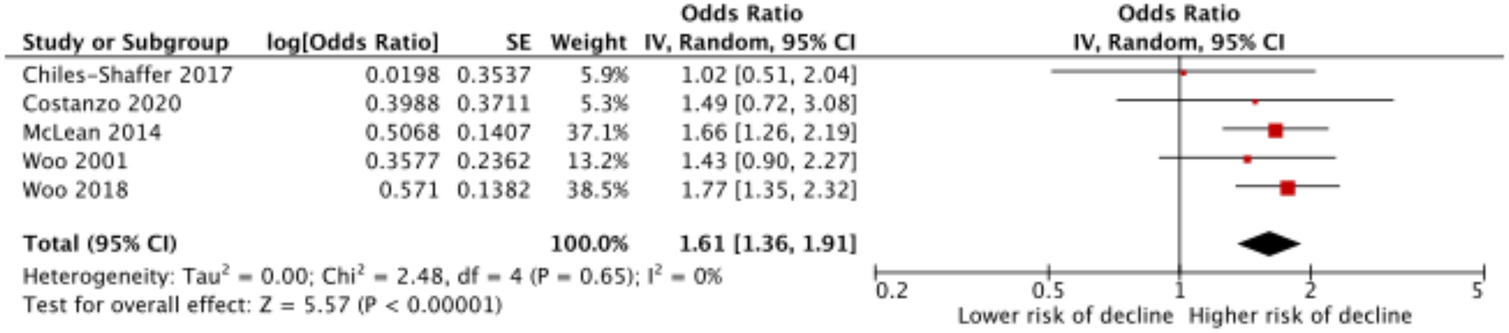

6a. Type of outcome measure: incident mobility limitations/disability (n=7)

Study or Subgroup	log[Odds Ratio]	SE	Weight	Odds Ratio IV, Random, 95% CI
Broadwin 2001	1.311	0.2928	11.9%	3.71 [2.09, 6.59]
Cawthon 2019	0.7655	0.2116	14.5%	2.15 [1.42, 3.26]
Cawthon 2020	-0.0202	0.0848	18.1%	0.98 [0.83, 1.16]
Delmonico 2007	0.1398	0.0764	18.3%	1.15 [0.99, 1.34]
Perez-Zepeda 2016	-0.2877	0.2494	13.2%	0.75 [0.46, 1.22]
Visser 1998	-0.3567	0.1616	16.1%	0.70 [0.51, 0.96]
Zoico 2007	-0.9676	0.4413	8.0%	0.38 [0.16, 0.90]
**Total (95% CI)**			**100.0%**	**1.12 [0.81, 1.55]**

Heterogeneity: $Tau^2 = 0.14$; $Chi^2 = 45.28$, df = 6 (P < 0.00001); $I^2 = 87\%$

Test for overall effect: Z = 0.70 (P = 0.48)

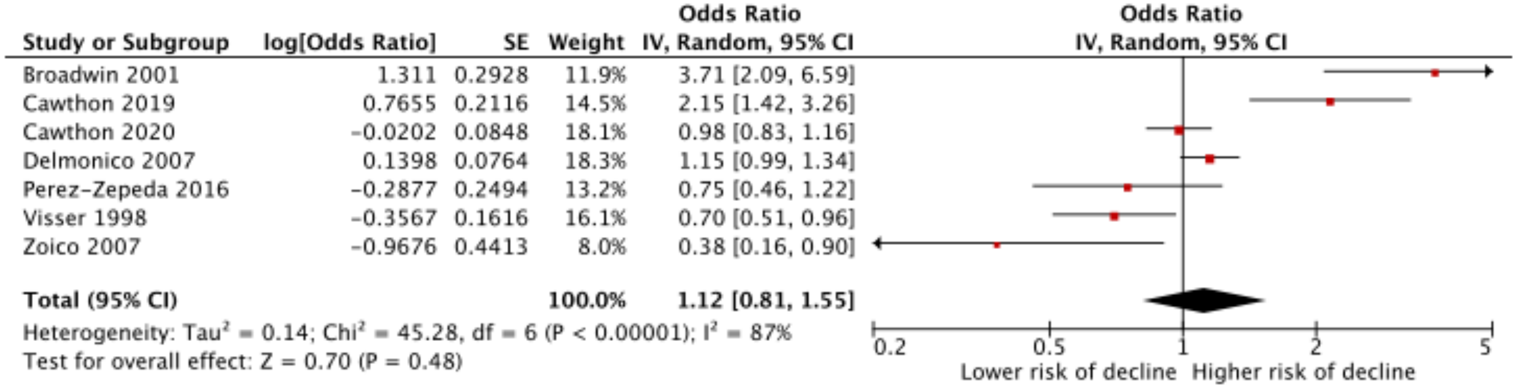

6a. Type of outcome measure: incident (i)ADL limitations/disability (n=19)

Study or Subgroup	log[Odds Ratio]	SE	Weight	Odds Ratio IV, Random, 95% CI
Alberti 2020	0.3148	0.1555	4.9%	1.37 [1.01, 1.86]
Amigues 2013	0.2546	0.1402	5.7%	1.29 [0.98, 1.70]
Baker 2018	0.207	0.0664	13.4%	1.23 [1.08, 1.40]
Broadwin 2001	1.1663	0.3521	1.2%	3.21 [1.61, 6.40]
Cawthon 2015	0.2624	0.109	8.1%	1.30 [1.05, 1.61]
Chiba 2021	0.0677	0.2784	1.8%	1.07 [0.62, 1.85]
Davies 2022	0.27	0.1586	4.8%	1.31 [0.96, 1.79]
Hirani 2015	1.0188	0.386	1.0%	2.77 [1.30, 5.90]
Ishii 2020	0.01	0.1727	4.2%	1.01 [0.72, 1.42]
Jang 2018	0.5008	0.2306	2.5%	1.65 [1.05, 2.59]
Janssen 2006	0.239	0.0874	10.5%	1.27 [1.07, 1.51]
Kitamura 2021	−0.1054	0.2069	3.1%	0.90 [0.60, 1.35]
Oh 2023	0.2776	0.2246	2.7%	1.32 [0.85, 2.05]
Rossi 2020	−0.0305	0.145	5.5%	0.97 [0.73, 1.29]
Seino 2022	0.1133	0.0629	14.0%	1.12 [0.99, 1.27]
Shimada 2021	0.0953	0.0857	10.7%	1.10 [0.93, 1.30]
Woo 2001	0.239	0.2422	2.3%	1.27 [0.79, 2.04]
Woo 2009	0.0296	0.2271	2.6%	1.03 [0.66, 1.61]
Zoico 2007	0.01	0.3587	1.1%	1.01 [0.50, 2.04]
**Total (95% CI)**			**100.0%**	**1.21 [1.12, 1.31]**

Heterogeneity: $Tau^2 = 0.01$; $Chi^2 = 25.21$, $df = 18$ ($P = 0.12$); $I^2 = 29\%$

Test for overall effect: $Z = 4.86$ ($P < 0.00001$)

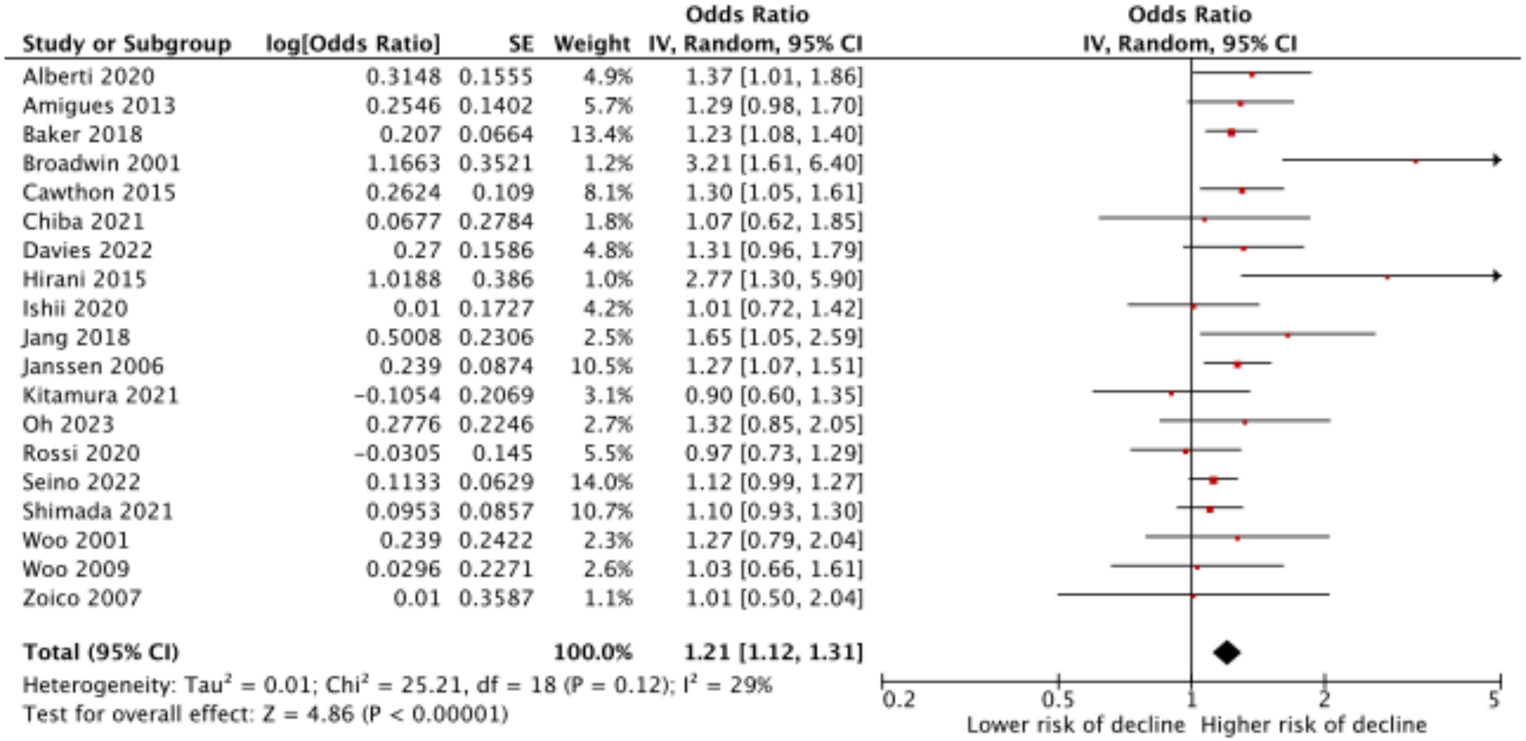

6b. Accuracy of body composition method to assess muscle mass: higher accurate methods DXA, D3Cr and CT (n=14)

Study or Subgroup	log[Odds Ratio]	SE	Weight	Odds Ratio IV, Random, 95% CI
Amigues 2013	0.2546	0.1402	8.4%	1.29 [0.98, 1.70]
Baker 2018	0.207	0.0664	11.3%	1.23 [1.08, 1.40]
Cawthon 2019	0.7655	0.2116	5.9%	2.15 [1.42, 3.26]
Cawthon 2020	−0.0202	0.0848	10.6%	0.98 [0.83, 1.16]
Chiles-Shaffer 2017	0.0198	0.3537	3.0%	1.02 [0.51, 2.04]
Davies 2022	0.27	0.1586	7.7%	1.31 [0.96, 1.79]
Delmonico 2007	0.1398	0.0764	10.9%	1.15 [0.99, 1.34]
Hirani 2015	1.0188	0.386	2.6%	2.77 [1.30, 5.90]
Ishii 2020	0.01	0.1727	7.1%	1.01 [0.72, 1.42]
McLean 2014	0.5068	0.1407	8.3%	1.66 [1.26, 2.19]
Oh 2023	0.2776	0.2246	5.5%	1.32 [0.85, 2.05]
Rossi 2020	−0.0305	0.145	8.2%	0.97 [0.73, 1.29]
Woo 2018	0.571	0.1382	8.4%	1.77 [1.35, 2.32]
Zoico 2007	−0.9676	0.4413	2.1%	0.38 [0.16, 0.90]
**Total (95% CI)**			**100.0%**	**1.26 [1.10, 1.45]**

Heterogeneity: $Tau^2 = 0.04$; $Chi^2 = 42.42$, df = 13 ($P < 0.0001$); $I^2 = 69\%$
Test for overall effect: $Z = 3.33$ ($P = 0.0009$)

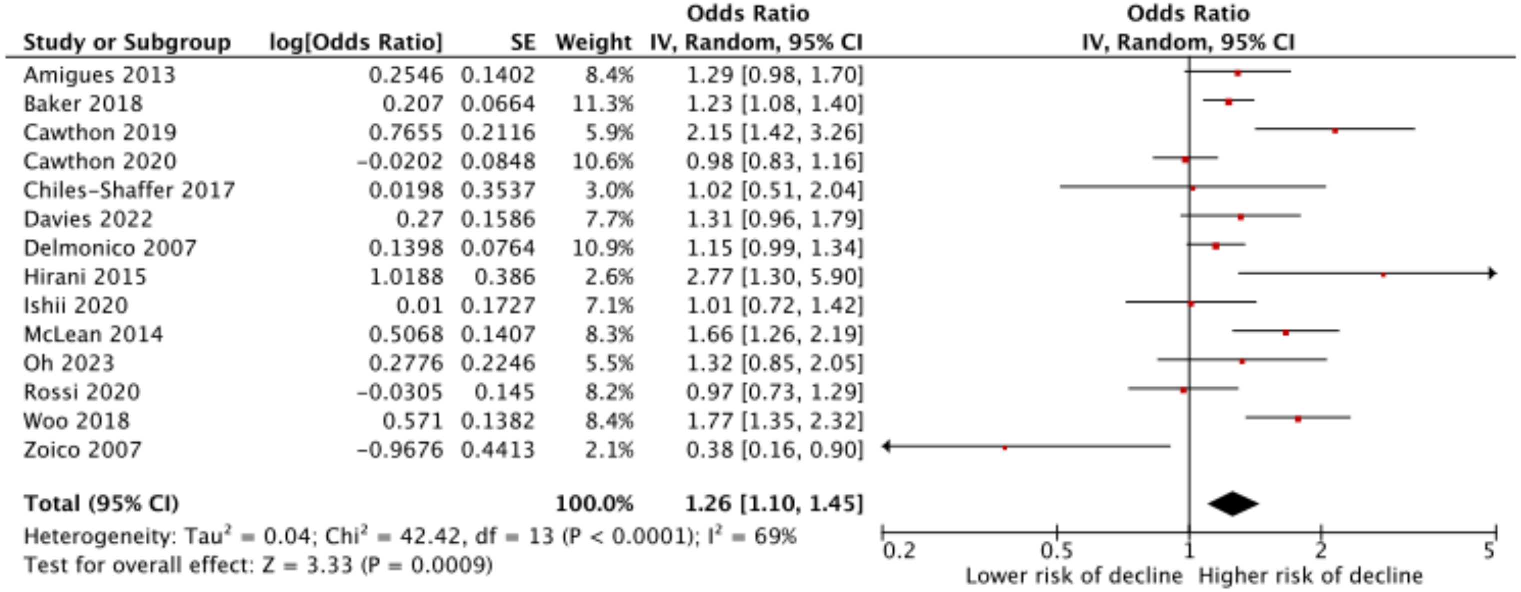

6b. Accuracy of body composition methods to assess muscle mass: lower accurate methods BIA and anthropometry (n=11)

Study or Subgroup	log[Odds Ratio]	SE	Weight	Odds Ratio IV, Random, 95% CI
Alberti 2020	0.3148	0.1555	11.0%	1.37 [1.01, 1.86]
Broadwin 2001	1.311	0.2928	6.3%	3.71 [2.09, 6.59]
Chiba 2021	0.0677	0.2784	6.7%	1.07 [0.62, 1.85]
Costanzo 2020	0.3988	0.3711	4.6%	1.49 [0.72, 3.08]
Jang 2018	0.5008	0.2306	8.1%	1.65 [1.05, 2.59]
Kitamura 2021	−0.1054	0.2069	9.0%	0.90 [0.60, 1.35]
Perez-Zepeda 2016	−0.2877	0.2494	7.5%	0.75 [0.46, 1.22]
Seino 2022	0.1133	0.0629	14.5%	1.12 [0.99, 1.27]
Shimada 2021	0.0953	0.0857	13.7%	1.10 [0.93, 1.30]
Visser 1998	−0.3567	0.1616	10.7%	0.70 [0.51, 0.96]
Woo 2001	0.3577	0.2362	7.9%	1.43 [0.90, 2.27]
**Total (95% CI)**			**100.0%**	**1.18 [0.98, 1.43]**

Heterogeneity: $Tau^2 = 0.06$; $Chi^2 = 35.11$, df = 10 (P = 0.0001); $I^2 = 72\%$
Test for overall effect: Z = 1.77 (P = 0.08)

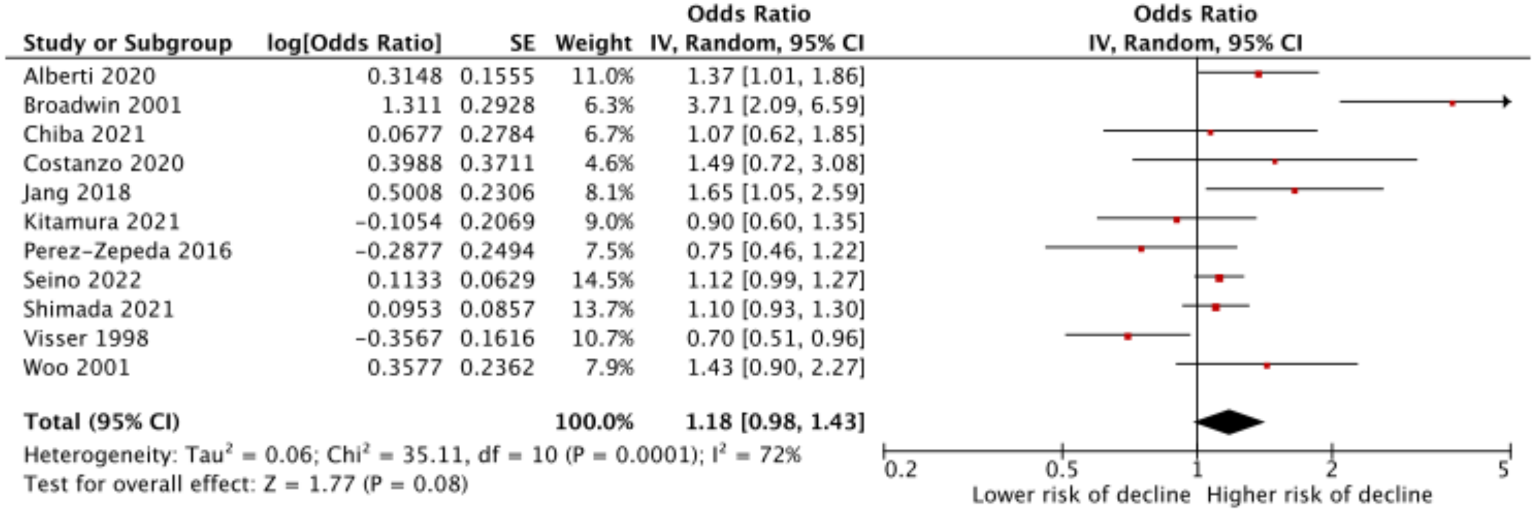

6c. Adjustment for body size: no adjustment or divided by height squared (n=18)

Study or Subgroup	log[Odds Ratio]	SE	Weight	Odds Ratio IV, Random, 95% CI
Alberto 2020	0.3148	0.1555	6.3%	1.37 [1.01, 1.86]
Baker 2020	0.1655	0.0948	7.5%	1.18 [0.98, 1.42]
Cawthon 2019	−0.1054	0.2069	5.2%	0.90 [0.60, 1.35]
Cawthon 2020	−0.0202	0.0848	7.7%	0.98 [0.83, 1.16]
Chiles Shaffer 2017	0.4318	0.3809	2.7%	1.54 [0.73, 3.25]
Costanzo 2020	0.3988	0.3711	2.8%	1.49 [0.72, 3.08]
Delmonico 2007	−0.2744	0.0877	7.7%	0.76 [0.64, 0.90]
Hirani 2015	0.3221	0.3249	3.4%	1.38 [0.73, 2.61]
Ishii 2020	0.01	0.1727	5.9%	1.01 [0.72, 1.42]
Jang 2018	0.2624	0.2414	4.6%	1.30 [0.81, 2.09]
Kitamura 2021	−0.1054	0.2069	5.2%	0.90 [0.60, 1.35]
McLean 2014	−0.0834	0.1618	6.2%	0.92 [0.67, 1.26]
Oh 2023	−0.0101	0.1992	5.4%	0.99 [0.67, 1.46]
Perez-Zepeda 2016	−0.2877	0.2494	4.5%	0.75 [0.46, 1.22]
Rossi 2020	−0.0305	0.145	6.5%	0.97 [0.73, 1.29]
Visser 1998	−0.3567	0.1616	6.2%	0.70 [0.51, 0.96]
Woo 2001	0.3577	0.2362	4.7%	1.43 [0.90, 2.27]
Woo 2016	0.6471	0.1033	7.4%	1.91 [1.56, 2.34]
**Total (95% CI)**			**100.0%**	**1.07 [0.92, 1.24]**

Heterogeneity: $Tau^2 = 0.07$; $Chi^2 = 67.63$, df = 17 ($P < 0.00001$); $I^2 = 75\%$
Test for overall effect: Z = 0.88 (P = 0.38)

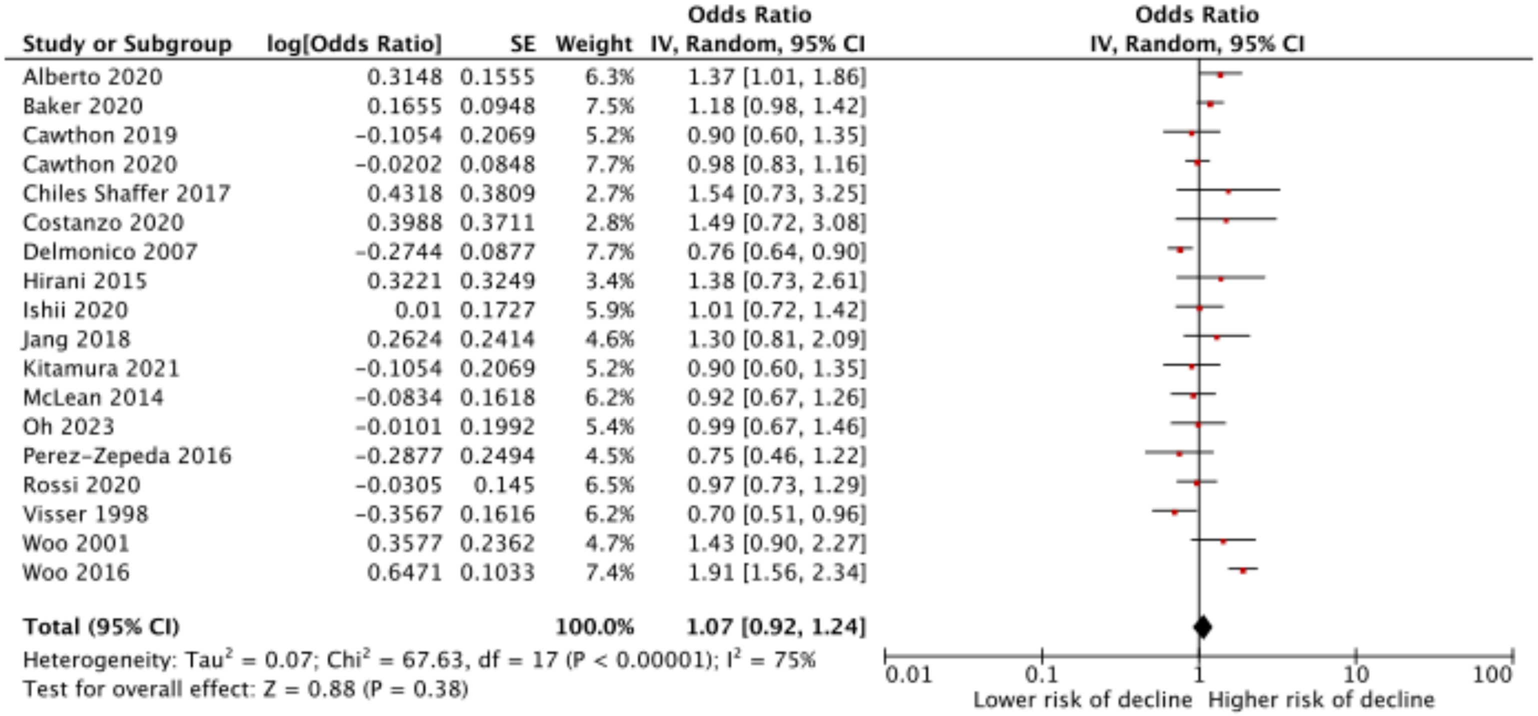

6c. Adjustment for body size: ratio of muscle mass divided by body weight, BMI or fat mass (n=10)

Study or Subgroup	log[Odds Ratio]	SE	Weight	Odds Ratio IV, Random, 95% CI
Amigues 2013	0.2546	0.1402	11.4%	1.29 [0.98, 1.70]
Baker 2018	-0.0619	0.1018	12.7%	0.94 [0.77, 1.15]
Baker 2020	0.2151	0.08	13.4%	1.24 [1.06, 1.45]
Broadwin 2001	1.311	0.2928	6.5%	3.71 [2.09, 6.59]
Cawthon 2019	0.7655	0.2116	8.9%	2.15 [1.42, 3.26]
Chiles-Shaffer 2017	0.0198	0.3537	5.2%	1.02 [0.51, 2.04]
Davies 2022	0.27	0.1586	10.7%	1.31 [0.96, 1.79]
Jang 2018	0.5008	0.2306	8.3%	1.65 [1.05, 2.59]
McLean 2014	0.5068	0.1407	11.4%	1.66 [1.26, 2.19]
Woo 2018	0.571	0.1382	11.5%	1.77 [1.35, 2.32]
**Total (95% CI)**			**100.0%**	**1.49 [1.22, 1.81]**

Heterogeneity: $Tau^2 = 0.07$; $Chi^2 = 38.04$, df = 9 (P < 0.0001); $I^2 = 76\%$
Test for overall effect: Z = 3.96 (P < 0.0001)

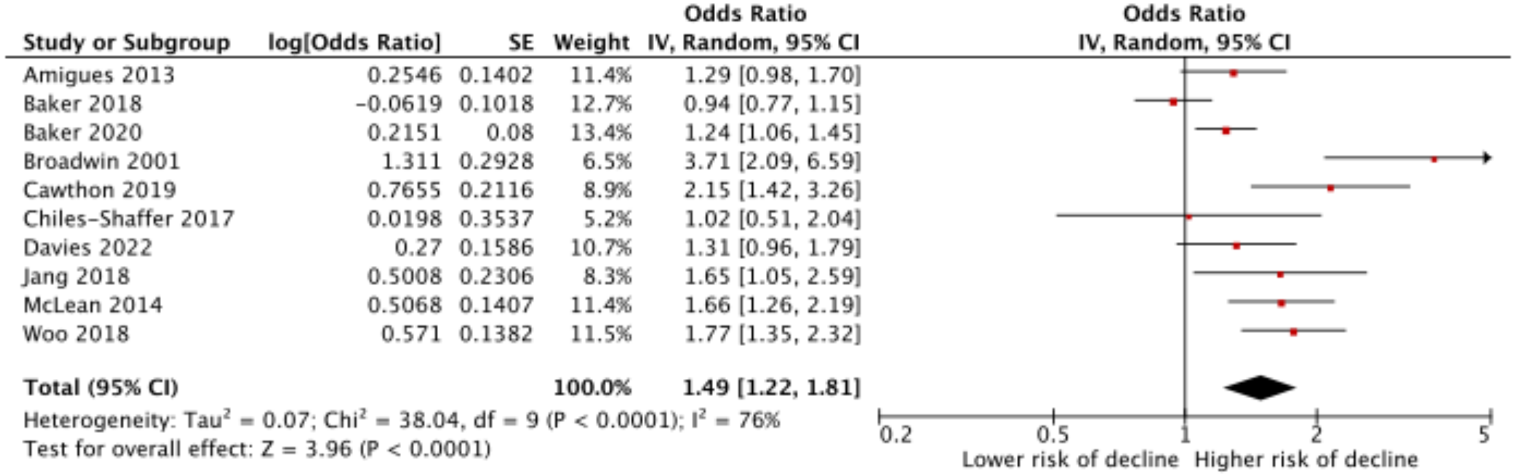

6c. Adjustment for body size: statistical adjustment for body weight, BMI or fat mass as confounders (n=11)

Study or Subgroup	log[Odds Ratio]	SE	Weight	Odds Ratio IV, Random, 95% CI
Baker 2018	0.207	0.0664	19.1%	1.23 [1.08, 1.40]
Cawthon 2019	0.1906	0.224	3.5%	1.21 [0.78, 1.88]
Chiba 2021	0.0677	0.2784	2.3%	1.07 [0.62, 1.85]
Delmonico 2007	0.1398	0.0764	16.7%	1.15 [0.99, 1.34]
Hirani 2015	1.0188	0.386	1.3%	2.77 [1.30, 5.90]
Janssen 2006	0.239	0.0874	14.5%	1.27 [1.07, 1.51]
Oh 2023	0.2776	0.2246	3.4%	1.32 [0.85, 2.05]
Seino 2022	0.1133	0.0629	20.0%	1.12 [0.99, 1.27]
Shimada 2021	0.0953	0.0857	14.8%	1.10 [0.93, 1.30]
Woo 2009	0.0296	0.2271	3.4%	1.03 [0.66, 1.61]
Zoico 2007	-0.9676	0.4413	1.0%	0.38 [0.16, 0.90]
**Total (95% CI)**			**100.0%**	**1.17 [1.07, 1.27]**

Heterogeneity: $Tau^2 = 0.01$; $Chi^2 = 14.71$, df = 10 (P = 0.14); $I^2 = 32\%$
Test for overall effect: Z = 3.56 (P = 0.0004)

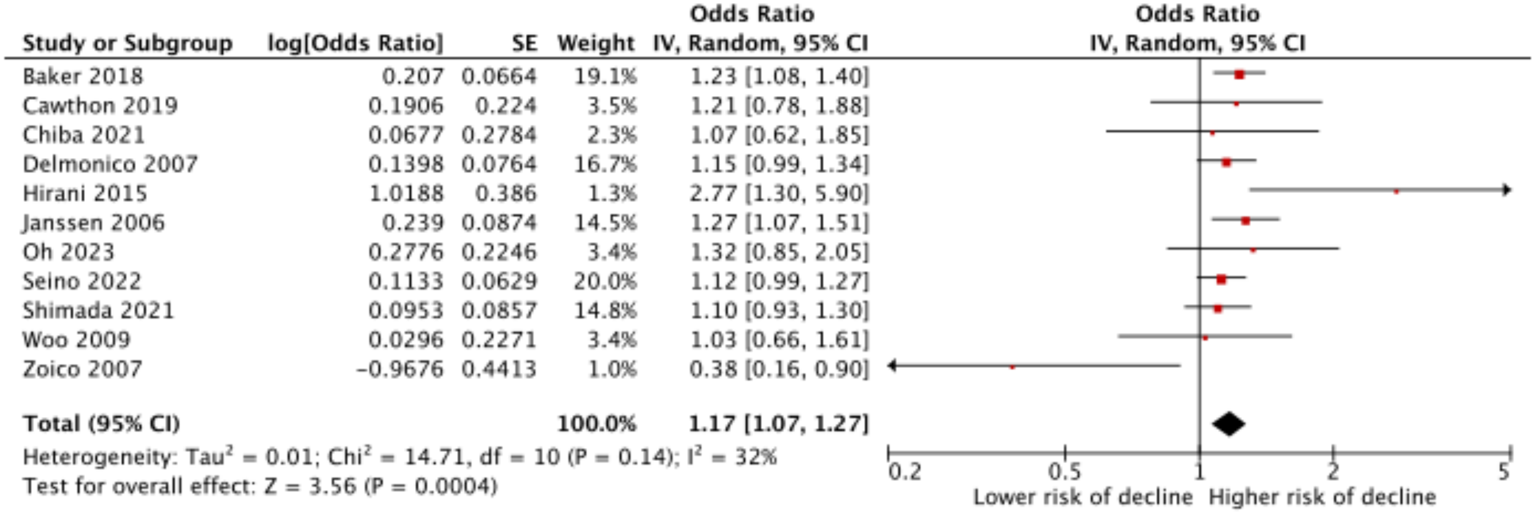

Annex 7. Random-effect meta-analysis of the risk of developing functional decline in older adults per standard deviation higher baseline muscle mass, categorized by a) type of outcome measure, b) accuracy of the body composition method to assess muscle mass, and c) adjustment for body size.

7a. Type of outcome measure: incident mobility limitations/disability (n=7)

Study or Subgroup	log[Odds Ratio]	SE	Weight	Odds Ratio IV, Random, 95% CI
Auyeung 2013	−0.1744	0.0444	21.4%	0.84 [0.77, 0.92]
Buchman 2021	0.0392	0.0738	18.9%	1.04 [0.90, 1.20]
Cawthon 2019	−0.3011	0.0741	18.9%	0.74 [0.64, 0.86]
Franzon 2019	0	0.305	4.8%	1.00 [0.55, 1.82]
Masugi 2022	−0.0101	0.1417	12.7%	0.99 [0.75, 1.31]
Nagae 2022	−0.0619	0.2376	7.0%	0.94 [0.59, 1.50]
Reinders 2015	−0.5108	0.1033	16.1%	0.60 [0.49, 0.73]
**Total (95% CI)**			**100.0%**	**0.84 [0.72, 0.97]**

Heterogeneity: $Tau^2 = 0.02$; $Chi^2 = 23.79$, df = 6 (P = 0.0006); $I^2 = 75\%$
Test for overall effect: Z = 2.32 (P = 0.02)

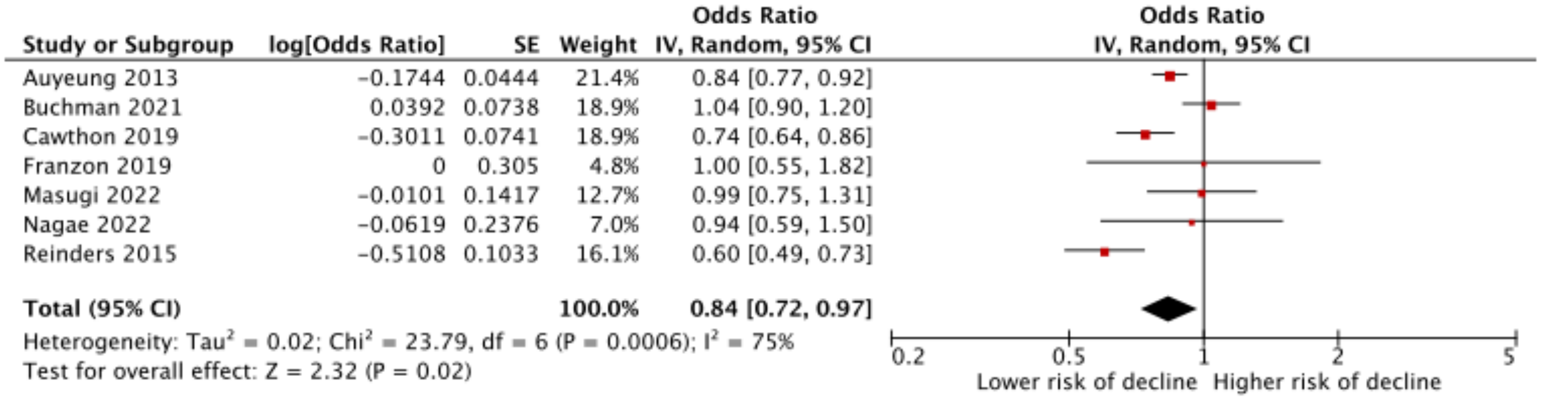

7a. Type of outcome measure: incident (i)ADL limitations/disability (n=12)

Study or Subgroup	log[Odds Ratio]	SE	Weight	Odds Ratio IV, Random, 95% CI
Amigues 2013	−0.1165	0.0481	12.6%	0.89 [0.81, 0.98]
Baker 2018	−0.0101	0.0264	13.8%	0.99 [0.94, 1.04]
Buchman 2021	0.1133	0.0578	11.9%	1.12 [1.00, 1.25]
Cawthon 2021	−0.3425	0.1211	7.6%	0.71 [0.56, 0.90]
Cesari 2015	−0.1278	0.0816	10.2%	0.88 [0.75, 1.03]
Fantin 2019	0.0198	0.1921	4.4%	1.02 [0.70, 1.49]
Franzon 2019	0.131	0.2636	2.8%	1.14 [0.68, 1.91]
Lam 2020	−0.2107	0.0393	13.1%	0.81 [0.75, 0.87]
Nagae 2022	−0.2614	0.3216	2.0%	0.77 [0.41, 1.45]
Tager 2004	−0.3857	0.1083	8.4%	0.68 [0.55, 0.84]
Uemura 2020	0.174	0.0939	9.4%	1.19 [0.99, 1.43]
Verlaan 2017	0.1222	0.216	3.8%	1.13 [0.74, 1.73]
**Total (95% CI)**			**100.0%**	**0.92 [0.84, 1.01]**

Heterogeneity: $Tau^2 = 0.02$; $Chi^2 = 51.68$, df = 11 ($P < 0.00001$); $I^2 = 79\%$
Test for overall effect: Z = 1.67 (P = 0.09)

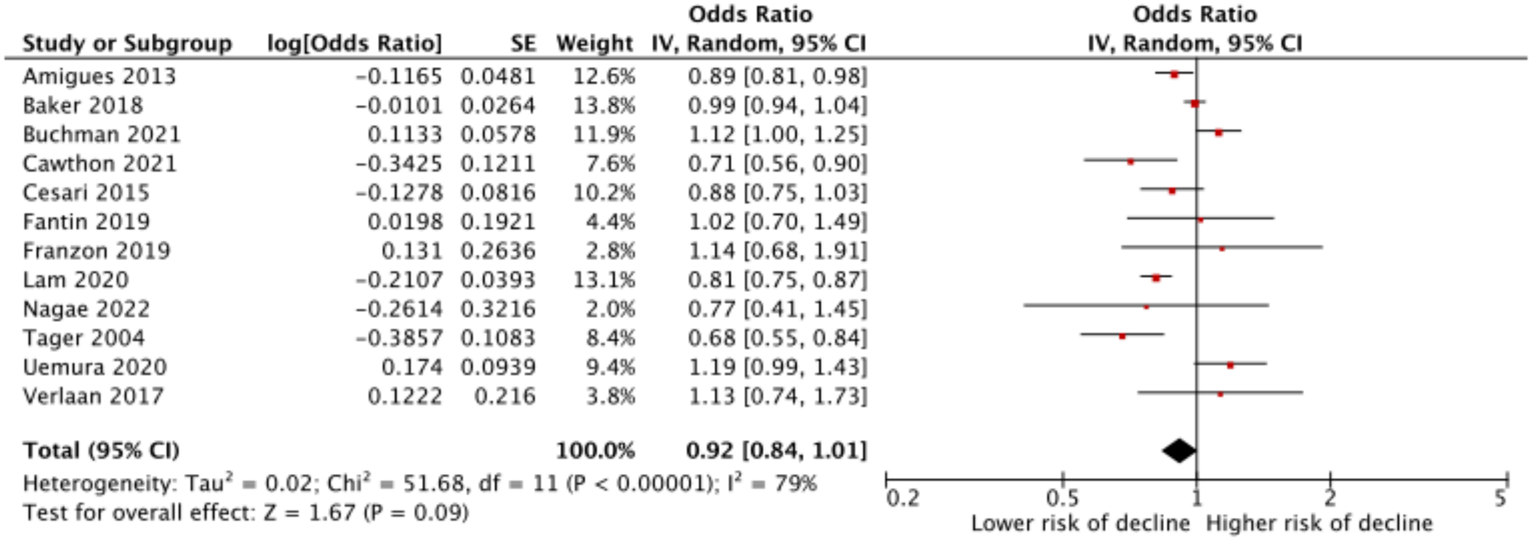

7b. Accuracy of body composition method to assess muscle mass: higher accurate methods DXA, D3Cr and CT (n=9)

Study or Subgroup	log[Odds Ratio]	SE	Weight	Odds Ratio IV, Random, 95% CI
Amigues 2013	−0.1165	0.0481	14.2%	0.89 [0.81, 0.98]
Auyeung 2013	−0.1744	0.0444	14.6%	0.84 [0.77, 0.92]
Baker 2018	−0.0101	0.0264	16.4%	0.99 [0.94, 1.04]
Cawthon 2019	−0.3011	0.0741	11.3%	0.74 [0.64, 0.86]
Cesari 2015	−0.1278	0.0816	10.5%	0.88 [0.75, 1.03]
Fantin 2017	0.0198	0.1921	3.7%	1.02 [0.70, 1.49]
Franzon 2019	0	0.305	1.7%	1.00 [0.55, 1.82]
Lam 2020	−0.2107	0.0393	15.2%	0.81 [0.75, 0.87]
Reinders 2015	−0.2614	0.0634	12.5%	0.77 [0.68, 0.87]
**Total (95% CI)**			**100.0%**	**0.86 [0.79, 0.93]**

Heterogeneity: $Tau^2 = 0.01$; $Chi^2 = 35.78$, df = 8 (P < 0.0001); $I^2 = 78\%$
Test for overall effect: Z = 3.72 (P = 0.0002)

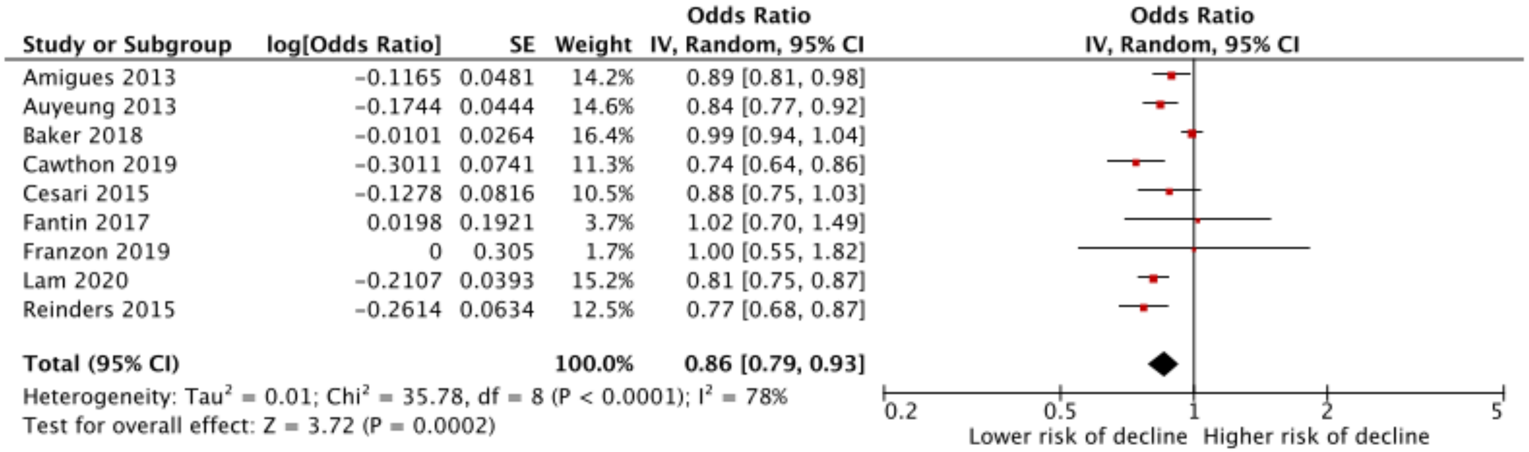

7b. Accuracy of body composition methods to assess muscle mass: lower accurate methods BIA and anthropometry (n=6)

Study or Subgroup	log[Odds Ratio]	SE	Weight	Odds Ratio IV, Random, 95% CI
Buchman 2021	0.0392	0.0738	22.6%	1.04 [0.90, 1.20]
Masugi 2022	−0.0101	0.1417	16.4%	0.99 [0.75, 1.31]
Nagae 2022	−0.0619	0.2376	9.8%	0.94 [0.59, 1.50]
Tager 2004	−0.3857	0.1083	19.4%	0.68 [0.55, 0.84]
Uemura 2020	0.174	0.0939	20.8%	1.19 [0.99, 1.43]
Verlaan 2017	0.1222	0.216	11.0%	1.13 [0.74, 1.73]
**Total (95% CI)**			**100.0%**	**0.98 [0.81, 1.17]**

Heterogeneity: $Tau^2 = 0.03$; $Chi^2 = 16.74$, df = 5 (P = 0.005); $I^2 = 70\%$
Test for overall effect: Z = 0.26 (P = 0.80)

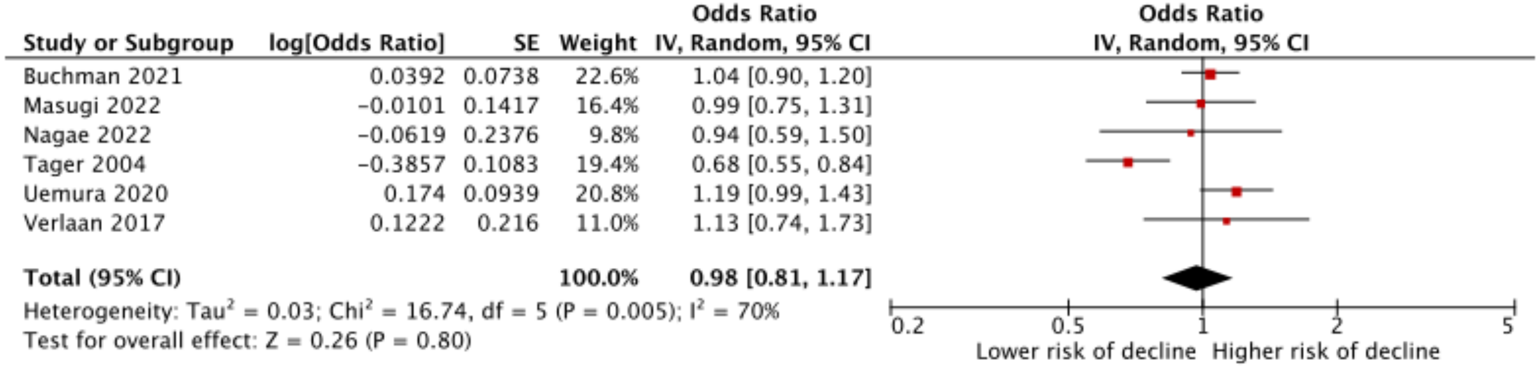

7c. Adjustment for body size: no adjustment or divided by height squared (n=8)

Study or Subgroup	log[Odds Ratio]	SE	Weight	Odds Ratio IV, Random, 95% CI
Amiques 2013	−0.1165	0.0481	21.3%	0.89 [0.81, 0.98]
Buchman 2021	0.0392	0.0738	15.7%	1.04 [0.90, 1.20]
Cawthon 2019	0.0296	0.0745	15.6%	1.03 [0.89, 1.19]
Cesari 2015	0.0583	0.0835	14.0%	1.06 [0.90, 1.25]
Fantin 2017	0.0198	0.1921	4.3%	1.02 [0.70, 1.49]
Lam 2020	0.1044	0.0431	22.5%	1.11 [1.02, 1.21]
Nagae 2022	−0.0619	0.2376	3.0%	0.94 [0.59, 1.50]
Verlaan 2017	−0.3147	0.2139	3.6%	0.73 [0.48, 1.11]
**Total (95% CI)**			**100.0%**	**1.01 [0.92, 1.09]**

Heterogeneity: $Tau^2 = 0.01$; $Chi^2 = 14.66$, df = 7 (P = 0.04); $I^2 = 52\%$
Test for overall effect: Z = 0.12 (P = 0.90)

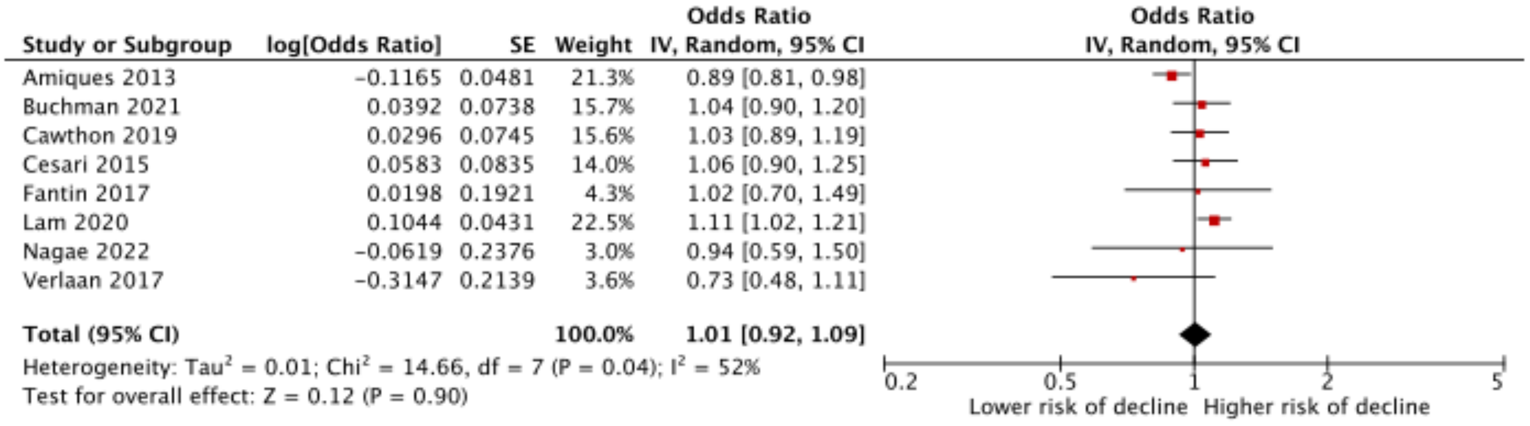

7c. Adjustment for body size: ratio of muscle mass divided by body weight, BMI or fat mass (n=5)

Study or Subgroup	log[Odds Ratio]	SE	Weight	Odds Ratio IV, Random, 95% CI
Auyeung 2013	−0.1744	0.0444	26.1%	0.84 [0.77, 0.92]
Cawthon 2019	−0.3011	0.0741	21.0%	0.74 [0.64, 0.86]
Lam 2020	−0.2107	0.0393	26.9%	0.81 [0.75, 0.87]
Tager 2004	−0.5108	0.0829	19.5%	0.60 [0.51, 0.71]
Verlaan 2017	0.1222	0.216	6.4%	1.13 [0.74, 1.73]
**Total (95% CI)**			**100.0%**	**0.77 [0.68, 0.87]**

Heterogeneity: $Tau^2 = 0.01$; $Chi^2 = 16.84$, df = 4 (P = 0.002); $I^2 = 76\%$
Test for overall effect: Z = 4.17 (P < 0.0001)

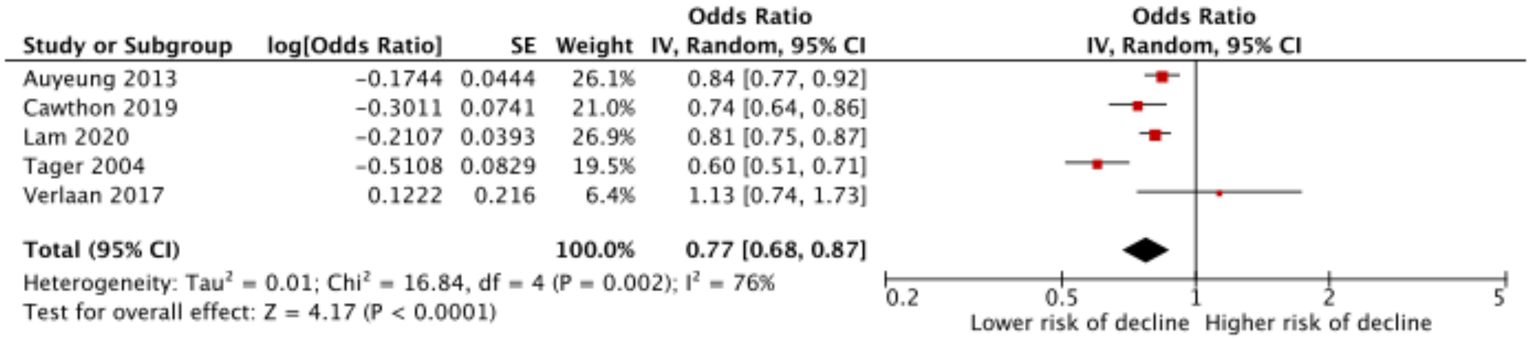

7c. Adjustment for body size: statistical adjustment for body weight, BMI or fat mass as confounders (n=8)

Study or Subgroup	log[Odds Ratio]	SE	Weight	Odds Ratio IV, Random, 95% CI
Baker 2018	−0.0101	0.0264	18.6%	0.99 [0.94, 1.04]
Cawthon 2019	−0.1054	0.0796	14.3%	0.90 [0.77, 1.05]
Cesari 2015	−0.1278	0.0816	14.1%	0.88 [0.75, 1.03]
Franzon 2019	0	0.305	3.1%	1.00 [0.55, 1.82]
Masugi 2022	−0.0101	0.1417	9.2%	0.99 [0.75, 1.31]
Reinders 2015	−0.2614	0.0634	15.8%	0.77 [0.68, 0.87]
Tager 2004	−0.3857	0.1083	11.7%	0.68 [0.55, 0.84]
Uemura 2020	0.174	0.0939	13.0%	1.19 [0.99, 1.43]
**Total (95% CI)**			**100.0%**	**0.90 [0.81, 1.02]**

Heterogeneity: $Tau^2 = 0.02$; $Chi^2 = 30.08$, df = 7 ($P < 0.0001$); $I^2 = 77\%$
Test for overall effect: Z = 1.69 (P = 0.09)

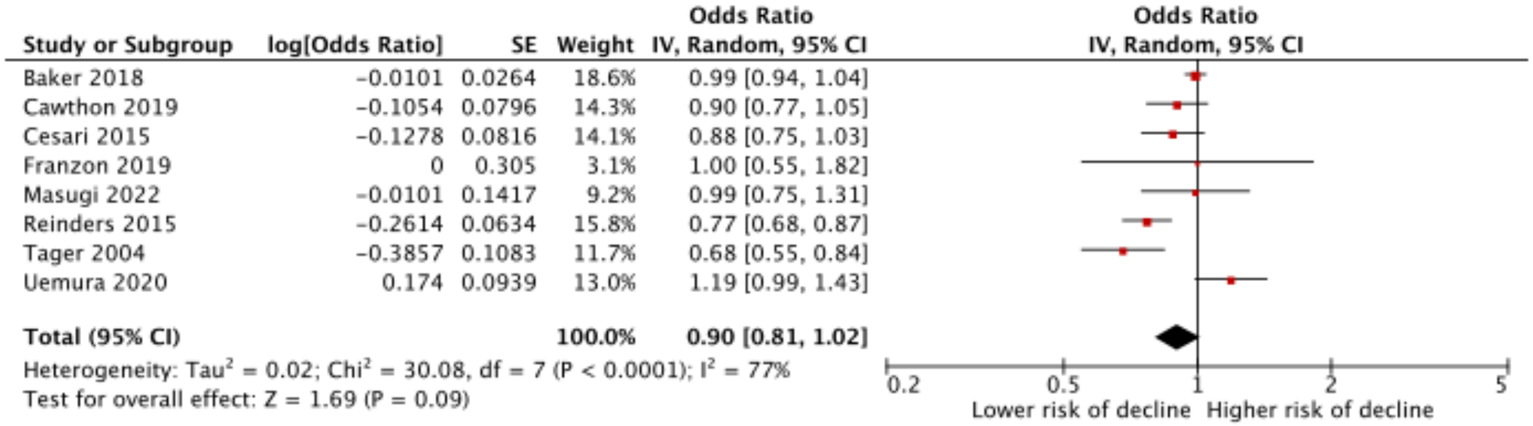

Annex 8. Overview of the eight studies investigating the association between baseline muscle mass and change in physical functioning that did not provide an effect size or used alternative methods to assess a potential association.

Reference	Year	Fat or weight adjustment	Description of association
Baumgartner	2004	Matched by obesity status	No effect size for muscle mass is provided. Text states that the hazard ratio for the sarcopenic-non-obese group was not statistically different from 1 using the non-sarcopenia-non-obese group as the reference group. Conclusion = no association.
Legrand	2014	BMI as confounder	The association is adjusted for grip strength as confounder. As grip strength is a potential mediator of the association, data were not extracted. Conclusion = NA.
Lera	2020	BMI as confounder	Study aim was to build a model to diagnose sarcopenia. Presented models are adjusted for sarcopenia status based on new model software, data were not extracted. Conclusion = NA.
Nagae	2022b (#142)	None	The t-test testing a difference in baseline BATT between those with and those without decline in ADL was not statistically significant (p=0.12). Conclusion = no association.
Wagner	2022	Fat mass/height squared as confounder	No effect sizes for the muscle mass parameters are provided. Text states that muscle mass was not included in the models, indicating that the muscle mass associations were not statistically significant. Conclusion = no association.
Zanker	2020	Using ratios	CART analyses were performed. A lower D3CrMM/wt was related to incident mobility limitations, but the DXA ALM parameters were not. Conclusion = significant association for D3Cr MM/wt only, not for the other muscle parameters.
Zanker	2022	Using ratios	Factor analysis was performed and only Factor 3, containing D3CrMM, strength and performance was associated with incident functional decline. As this factor also contains strength and performance, data were not extracted. Conclusion = NA.
Zhao	2024	BMI as confounder	Text states that the separate sarcopenia components, which include low versus not-low ALM/ht^2, were not associated with the onset of ADL and IADL. Conclusion = no association.

ADL=activities of daily living, NA=not applicable, CART=Classification And Regression Tree. For muscle mass parameter abbreviations, see table 2. For further study details, see Annex 3.
